# Supplementary material for: Improving the reliability of cohesion policy databases
Source: PLoS One. 2022 Apr 22;17(4):e0266823. doi: 10.1371/journal.pone.0266823 (PMC9032402; doi:10.1371/journal.pone.0266823)
Supplement: S1 File — (PDF) [file pone.0266823.s001.pdf]

Supporting Information - Improving the reliability of cohesion policy databases

[1], Samuele Lo Piano*[1], Emanuele Borgonovo[2], Arnald Puy[3,4], Andrea Saltelli[5], John Walsh[6], and Daniele Vidoni[7]

[1] *School of the Built Environment, University of Reading, United Kingdom*
[2] *Department of Decision Sciences and BIDSA, Bocconi University, Milano, Italy*
[3] *Department of Ecology and Evolutionary Biology, Princeton University, New Jersey, USA*
[4] *Centre for the Study of the Sciences and the Humanities (SVT), University of Bergen, Norway*
[5] *Barcelona School of Management, Universitat Pompeu Fabra, Barcelona, Spain*
[6] *Directorate-General for Regional and Urban Policy, European Commission, Brussels, Belgium*
[7] *Directorate-General for Competition, European Commission, Brussels, Belgium*

*Corresponding Author: s.lopiano@reading.ac.uk

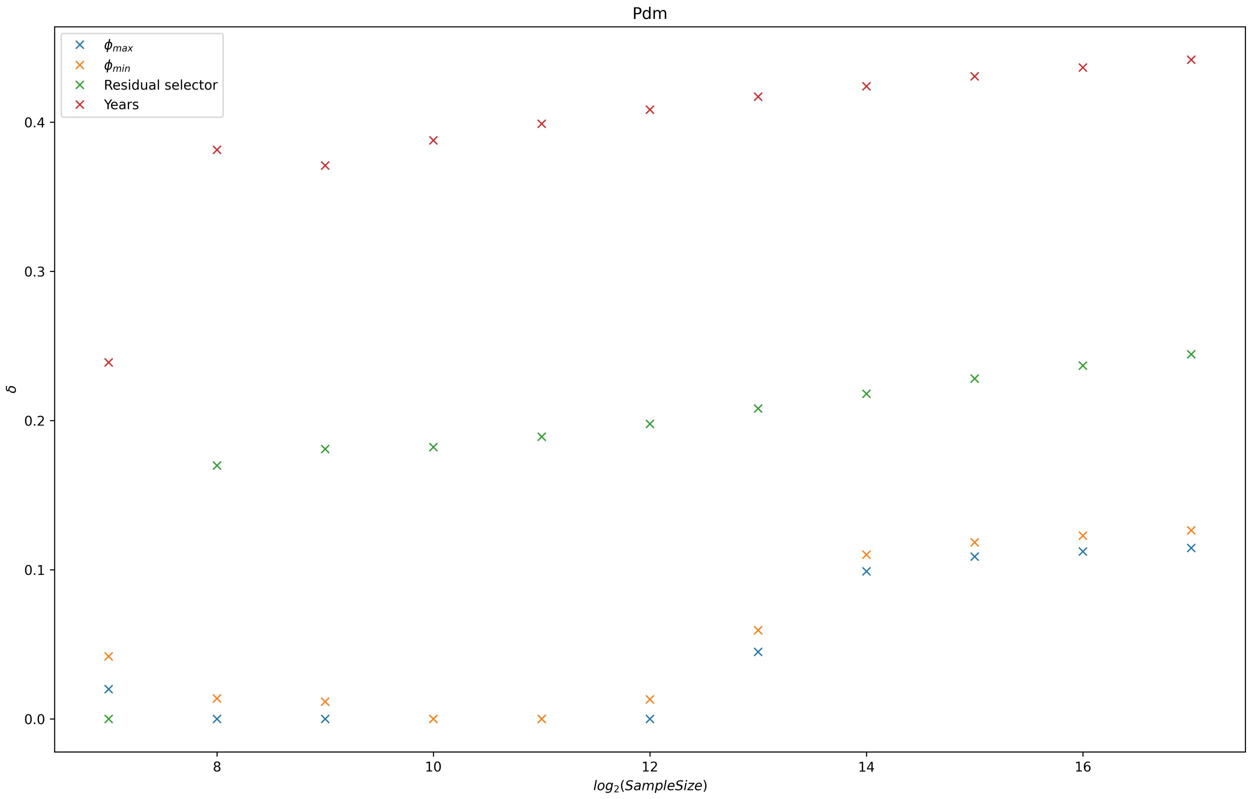

Fig. 1: Convergence plot for Pdm.

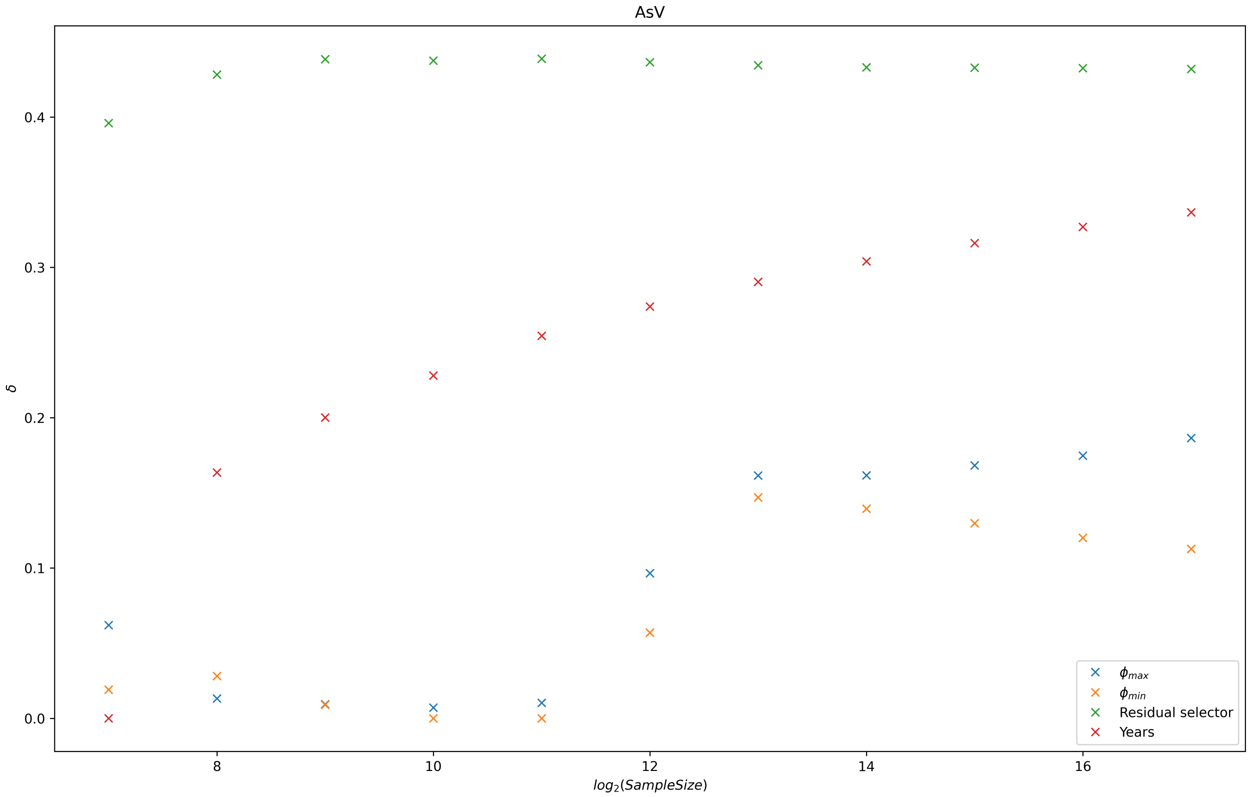

Fig. 2: Convergence plot for AsV.

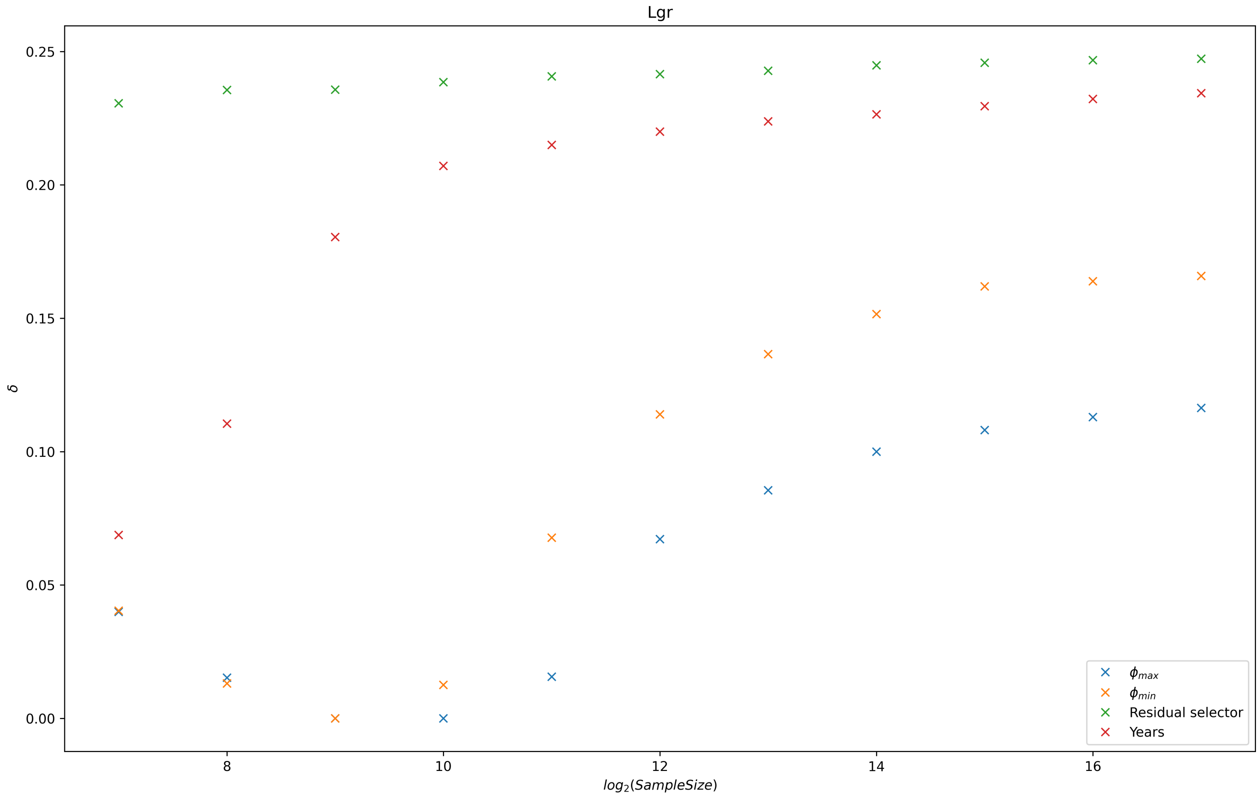

Fig. 3: Convergence plot for Lgr.

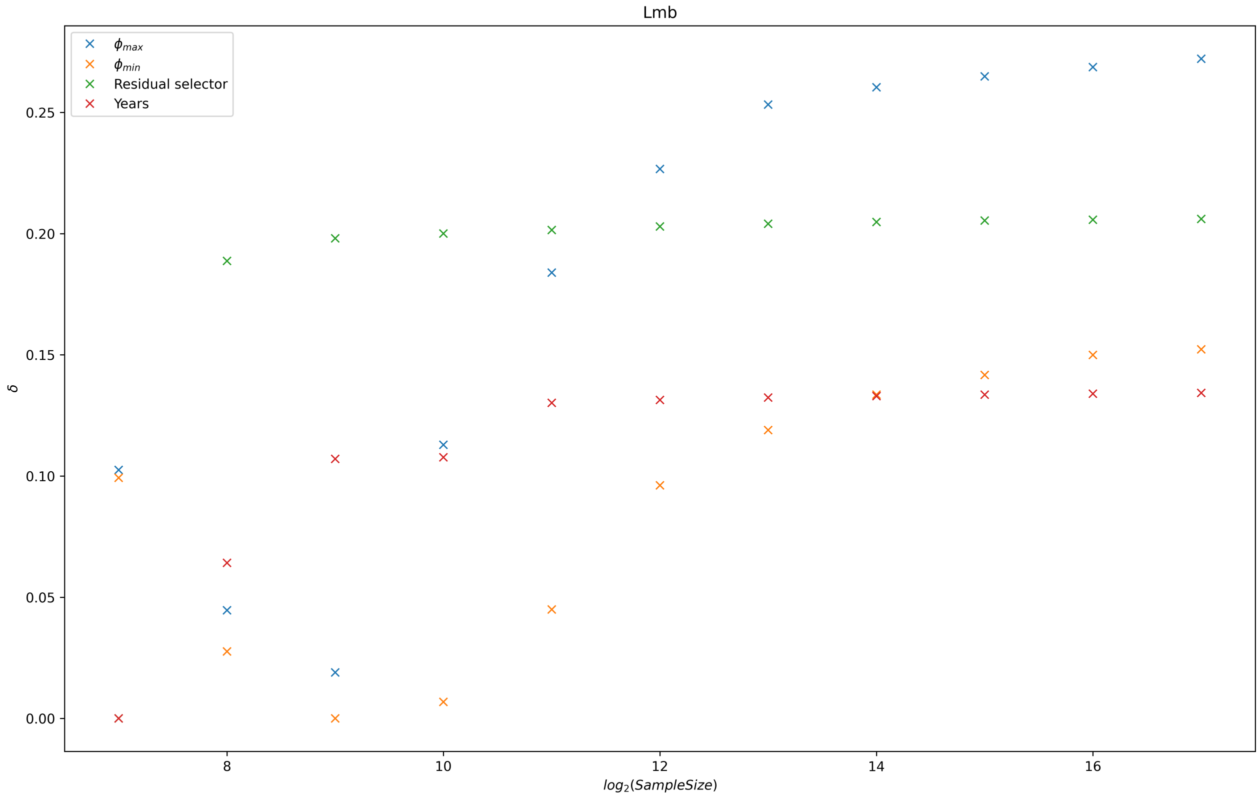

Fig. 4: Convergence plot for Lmb.

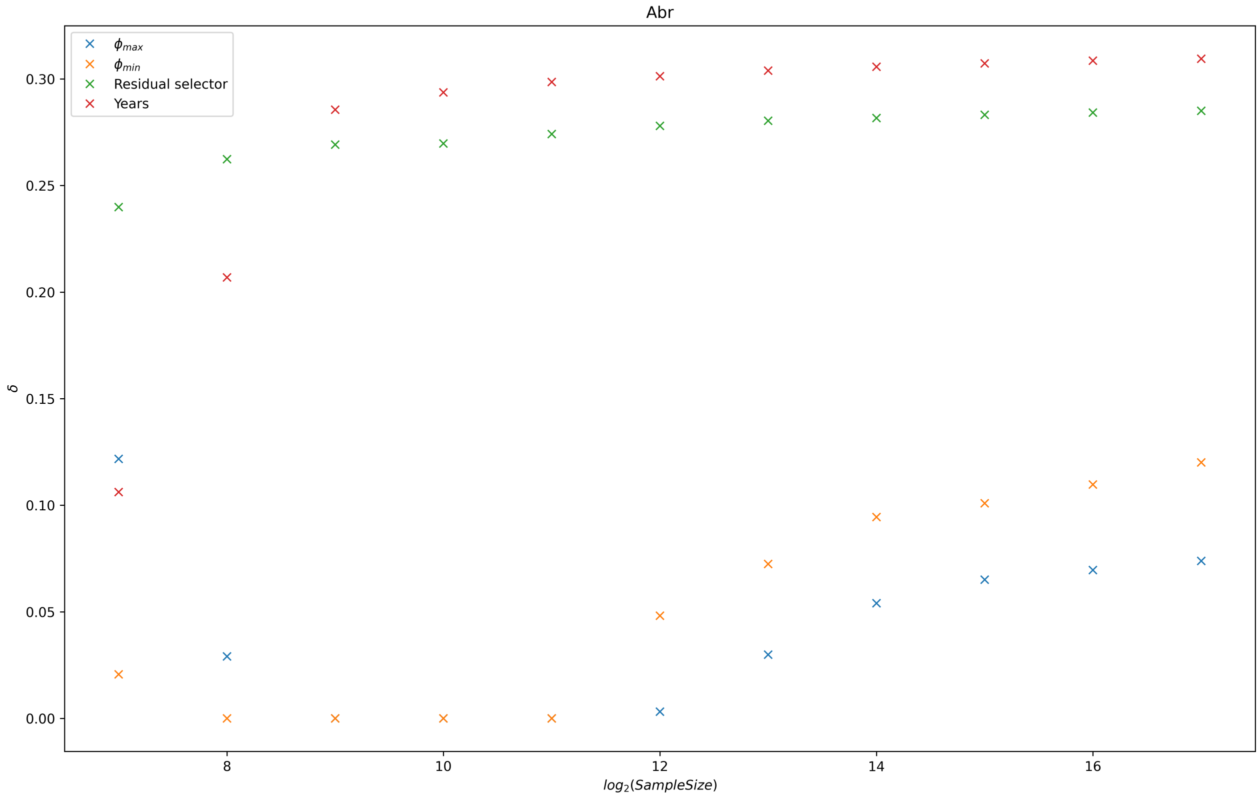

Fig. 5: Convergence plot for Abr.

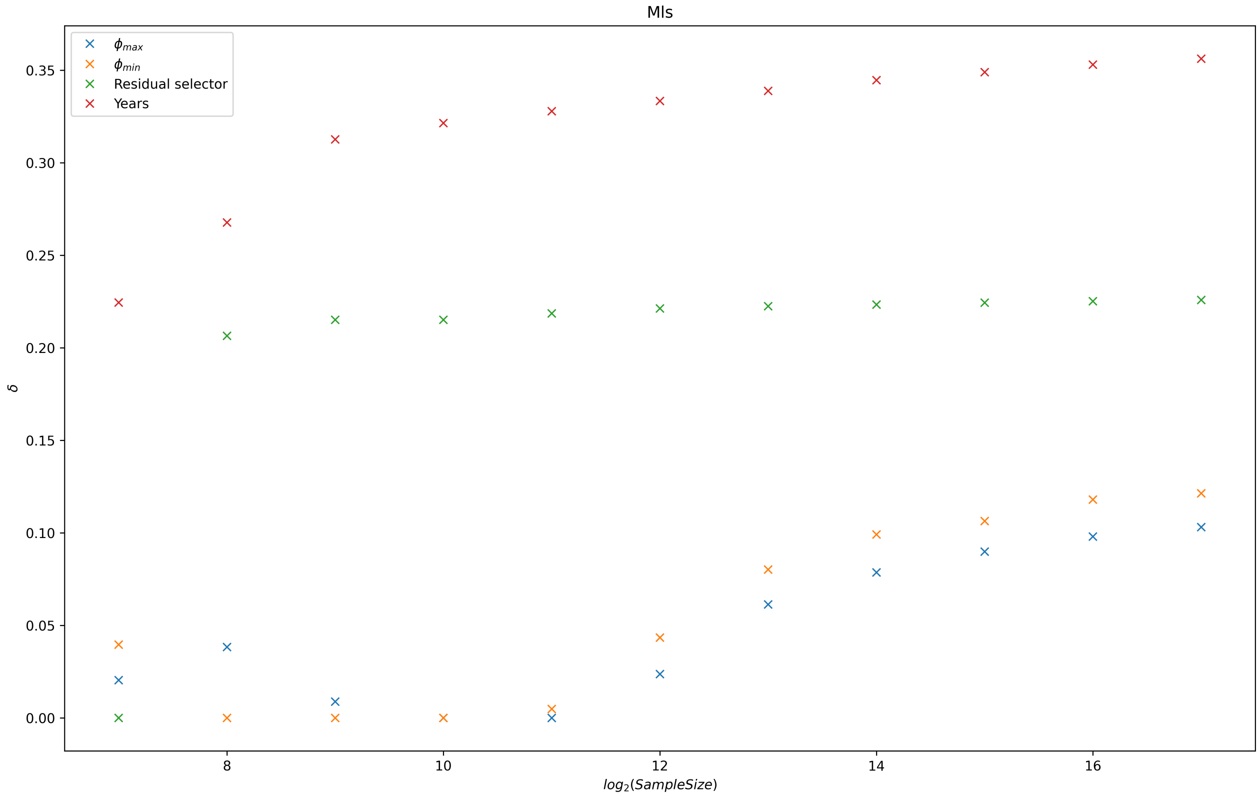

Fig. 6: Convergence plot for Mls.

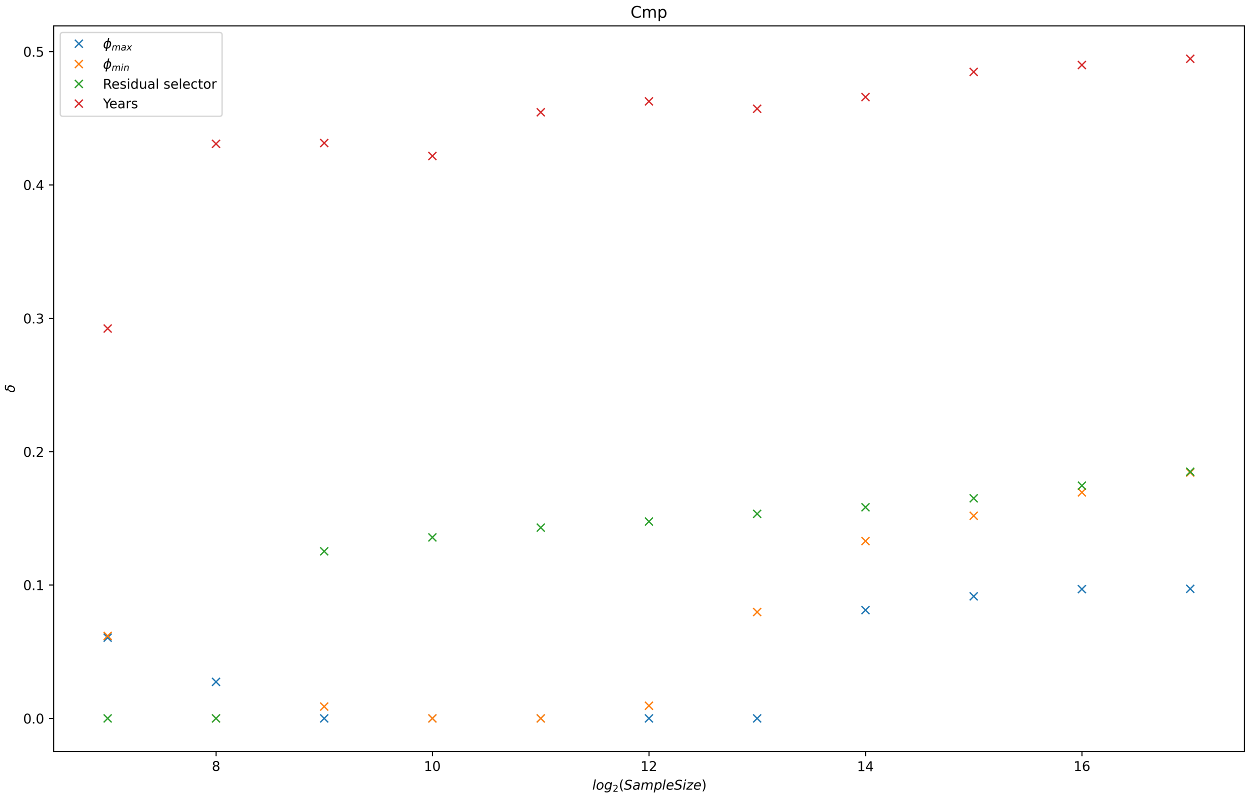

Fig. 7: Convergence plot for Cmp.

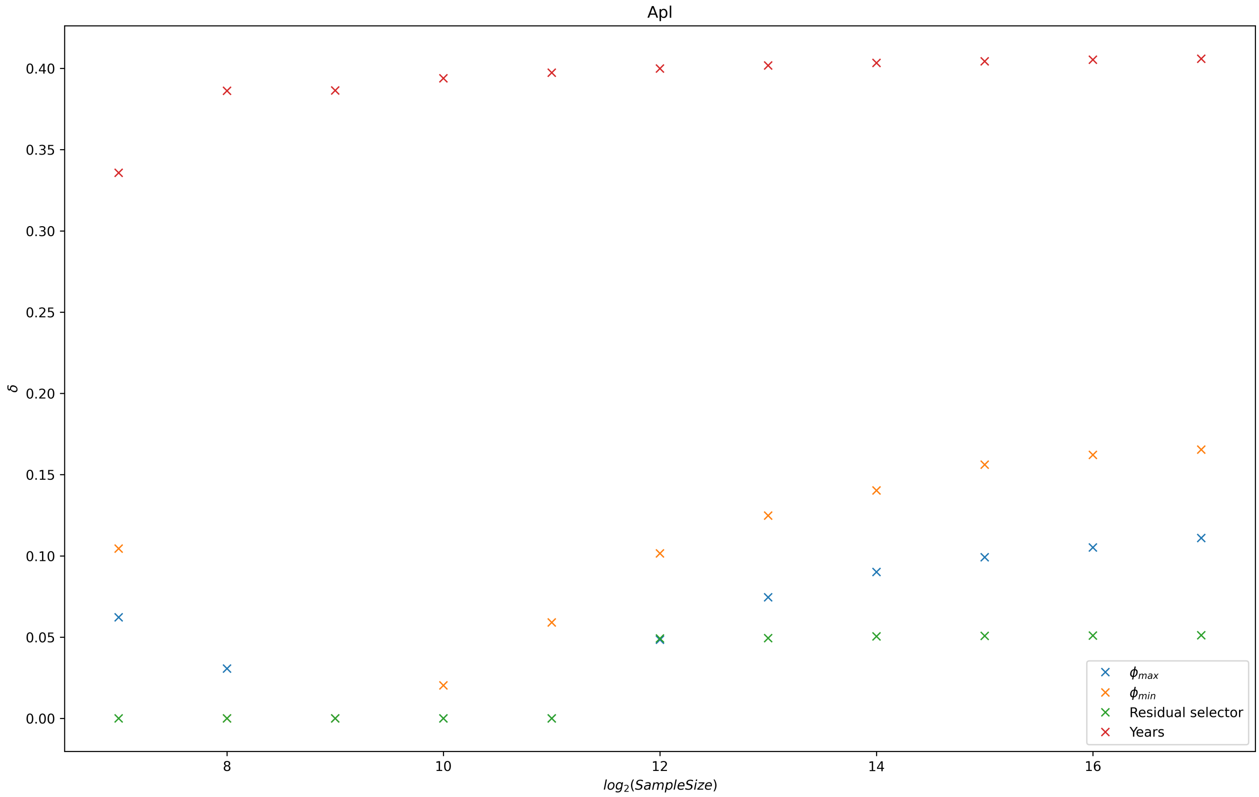

Fig. 8: Convergence plot for Apl.

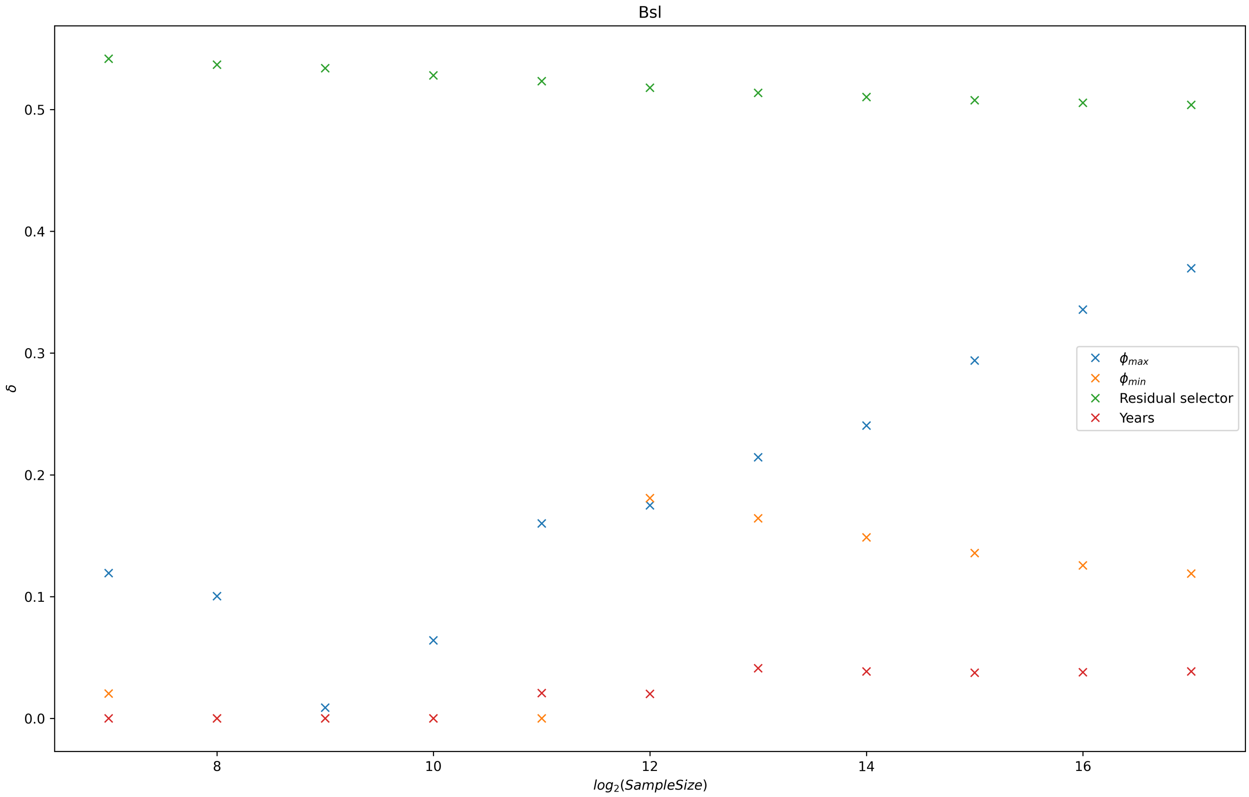

Fig. 9: Convergence plot for Bsl.

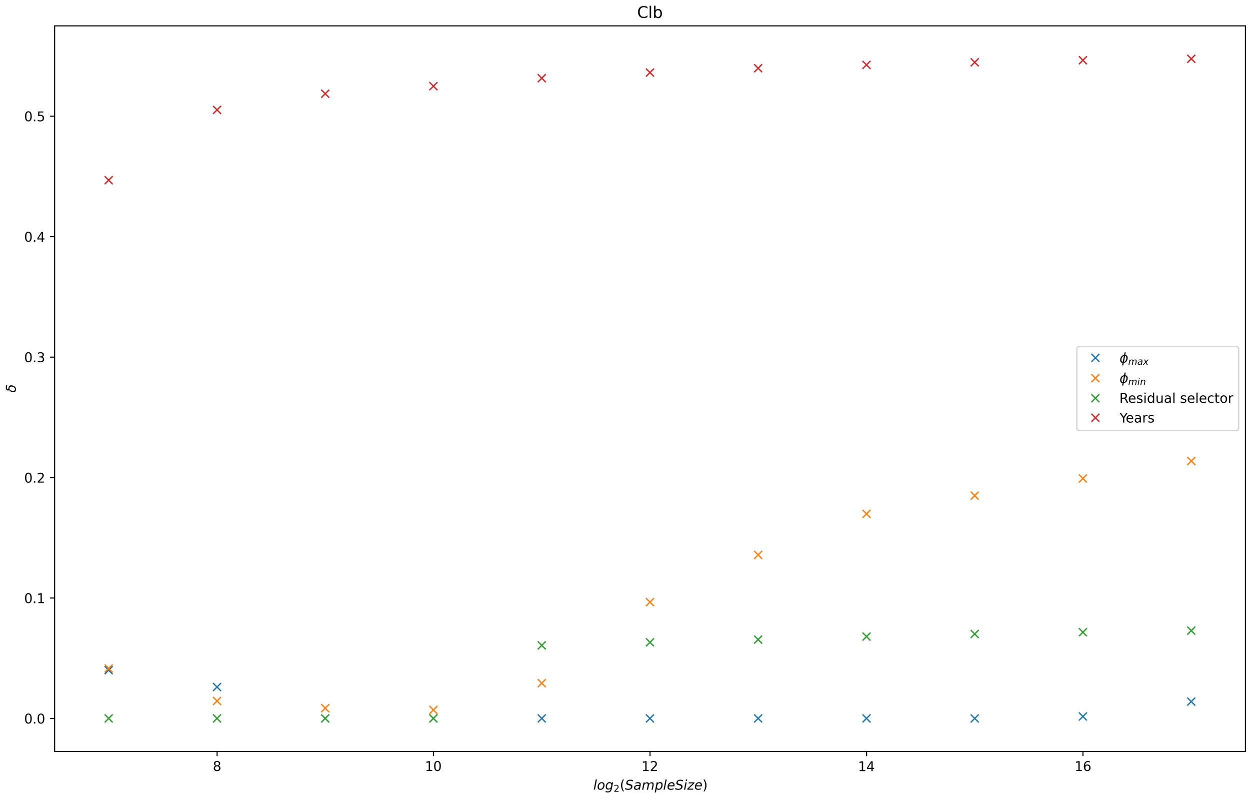

Fig. 10: Convergence plot for Clb.

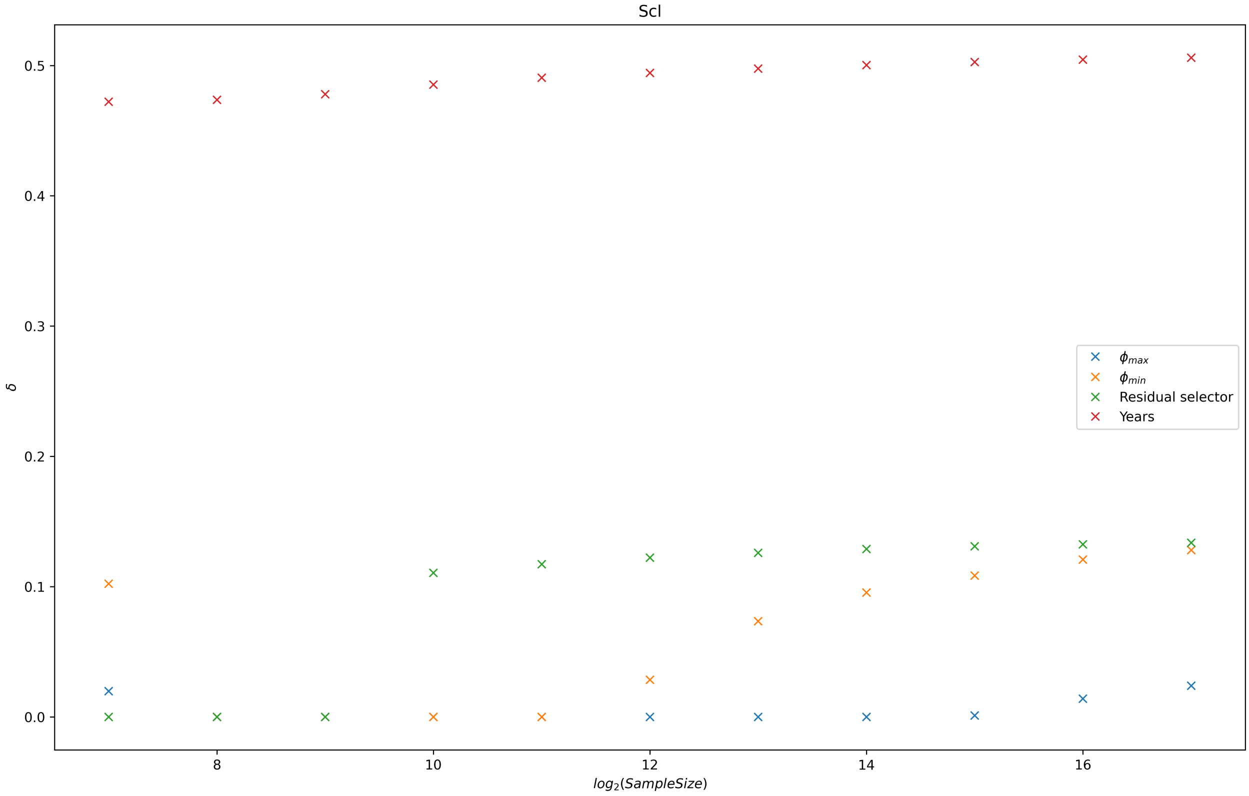

Fig. 11: Convergence plot for Scl.

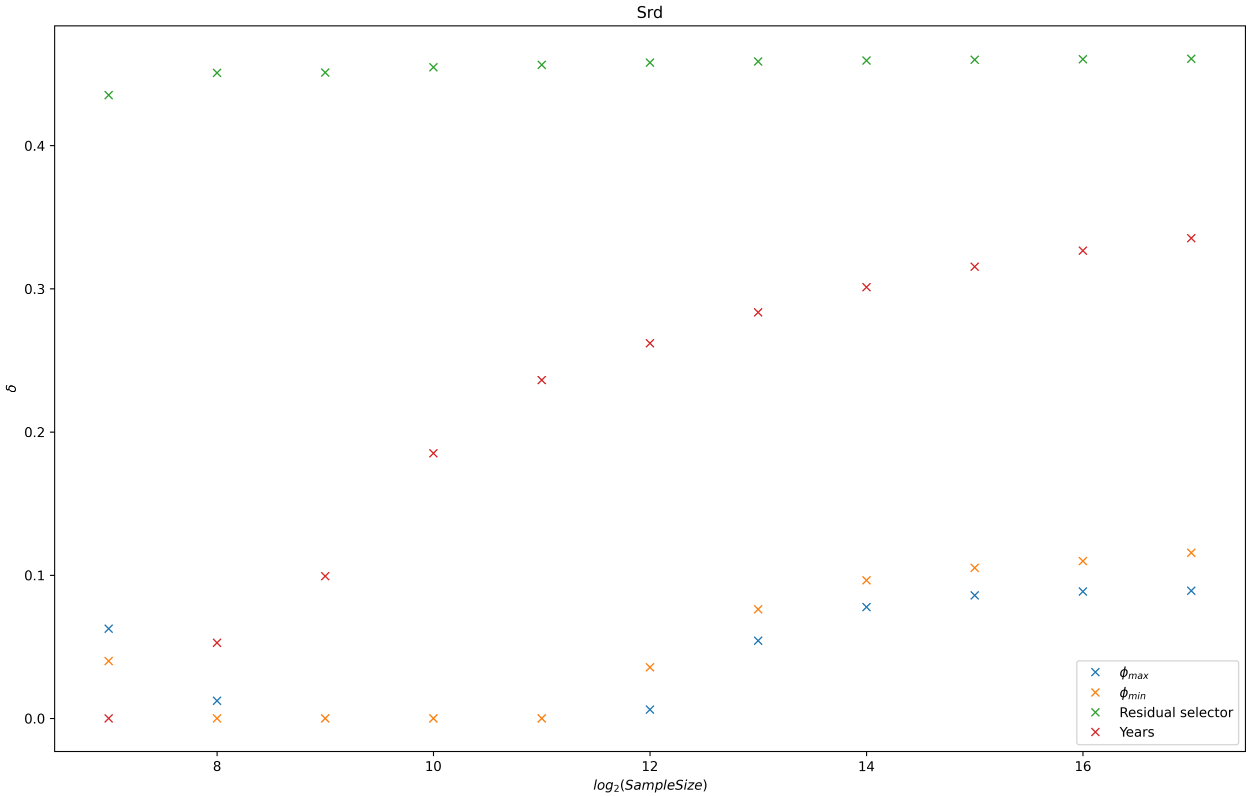

Fig. 12: Convergence plot for Srd.

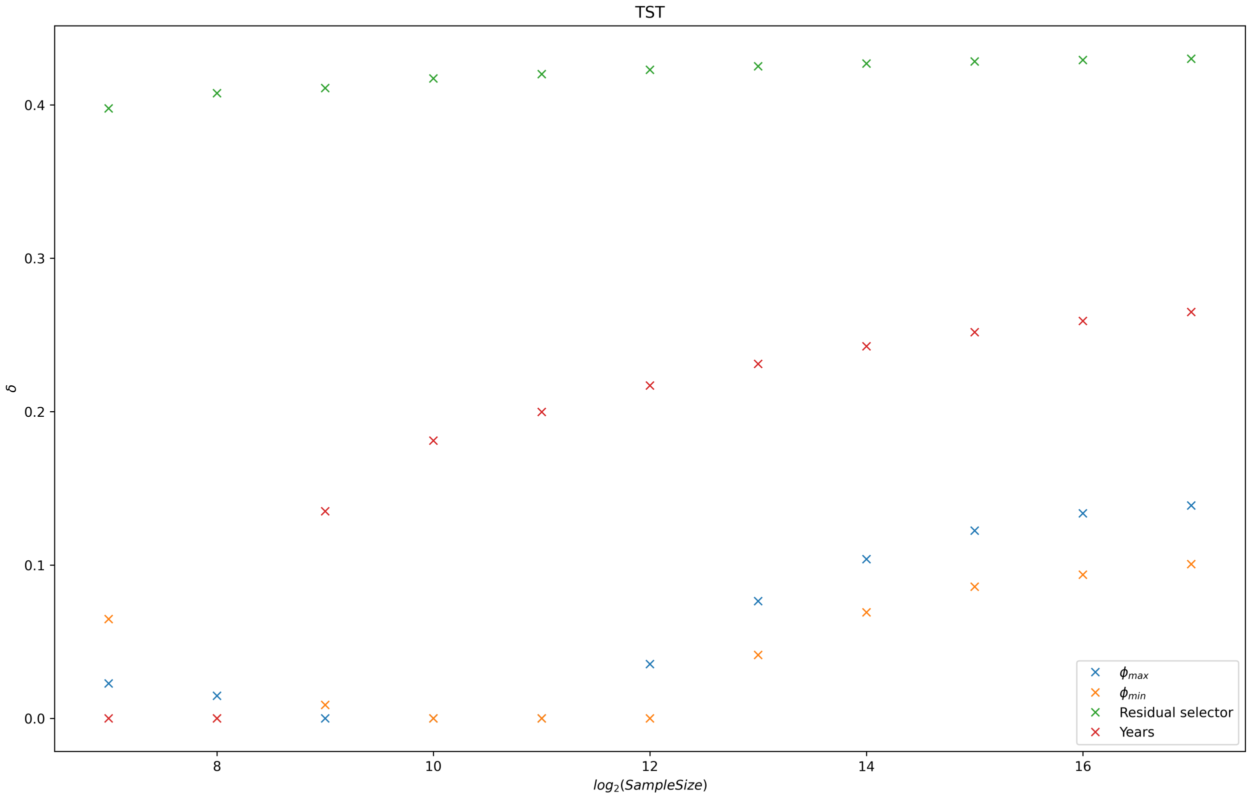

Fig. 13: Convergence plot for TST.

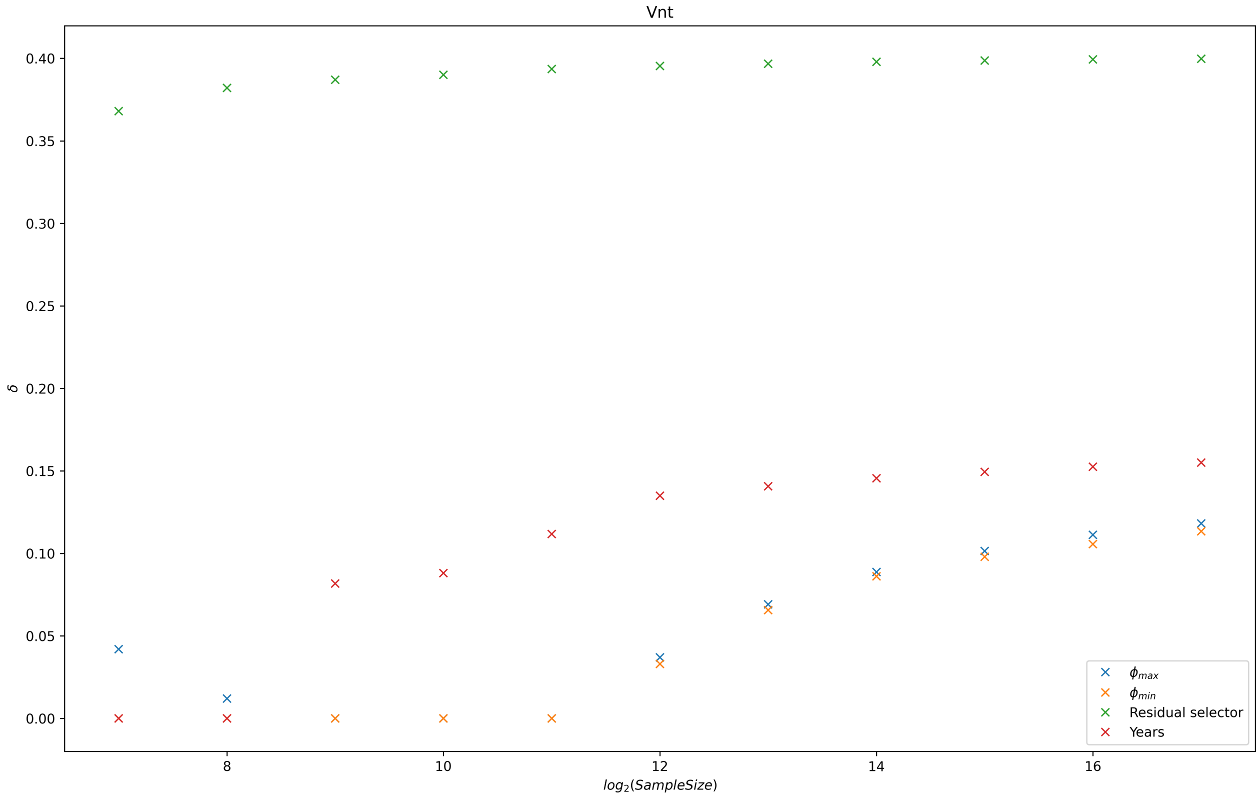

Fig. 14: Convergence plot for Vnt.

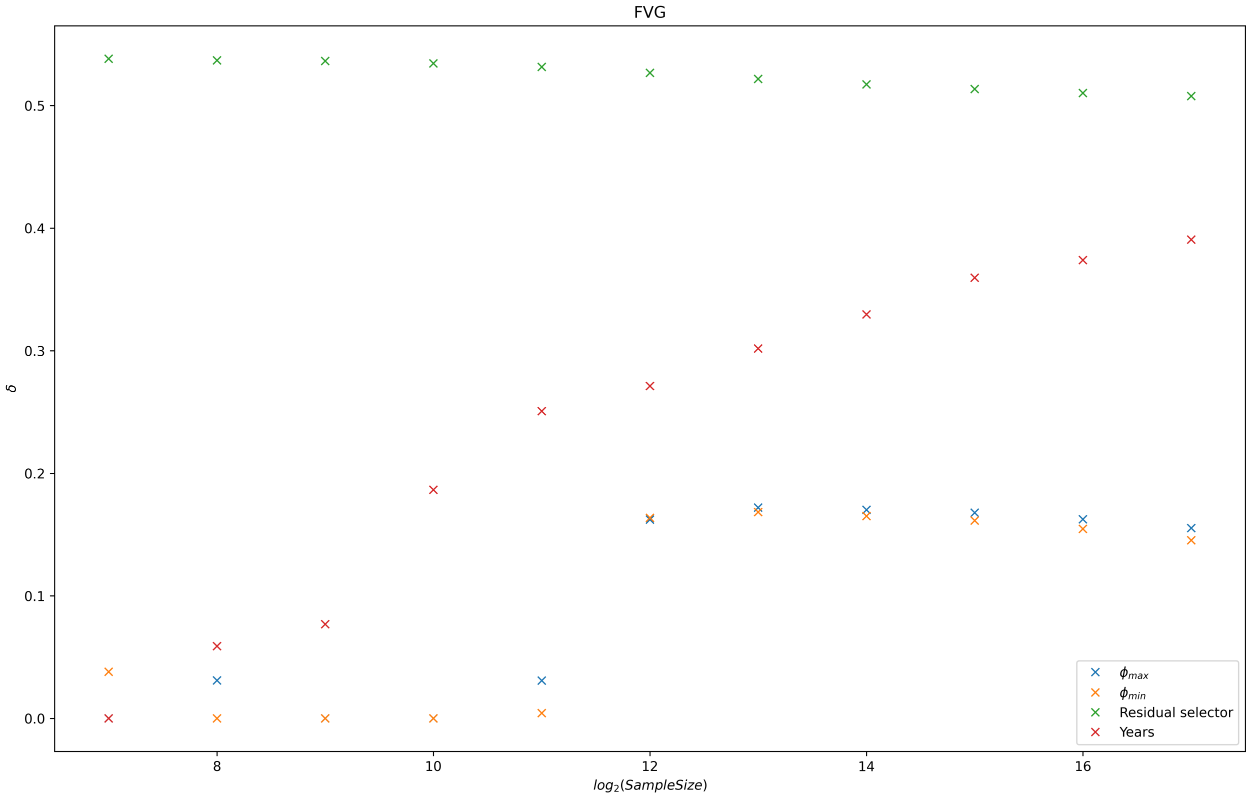

Fig. 15: Convergence plot for FVG.

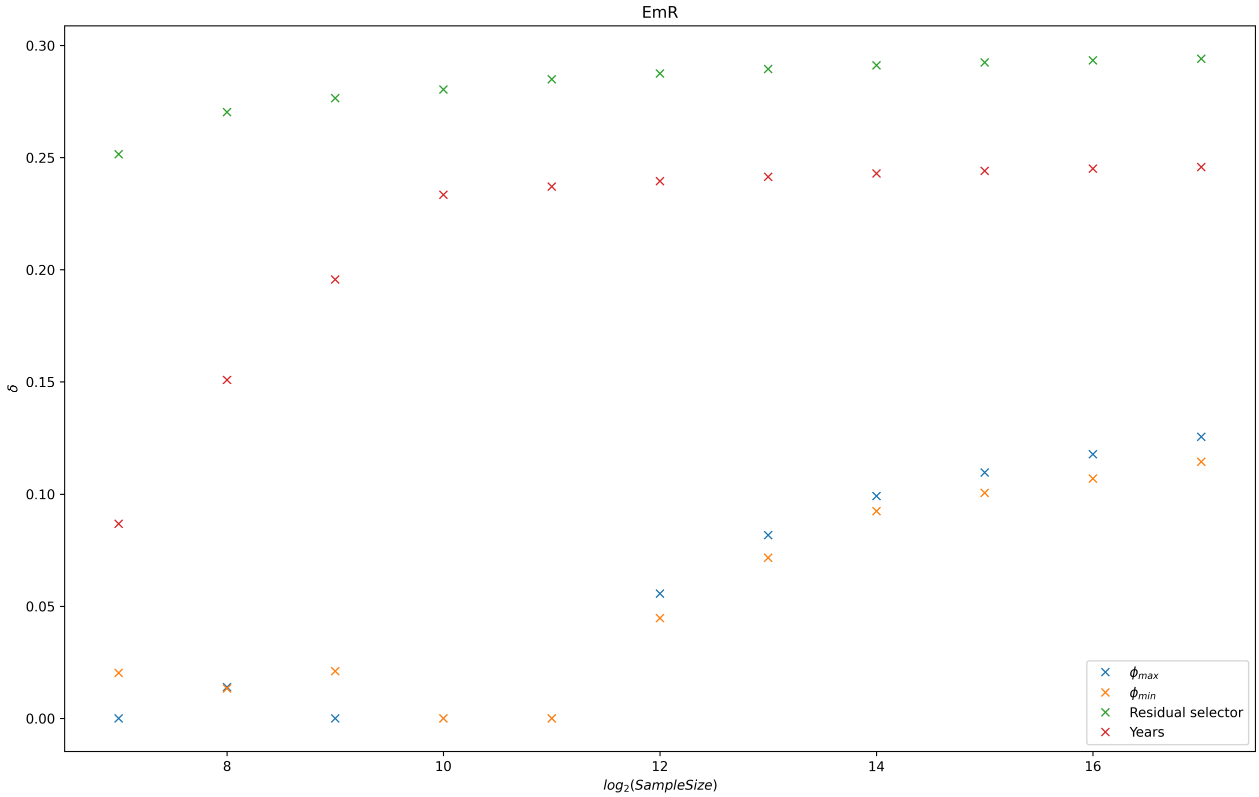

Fig. 16: Convergence plot for EmR.

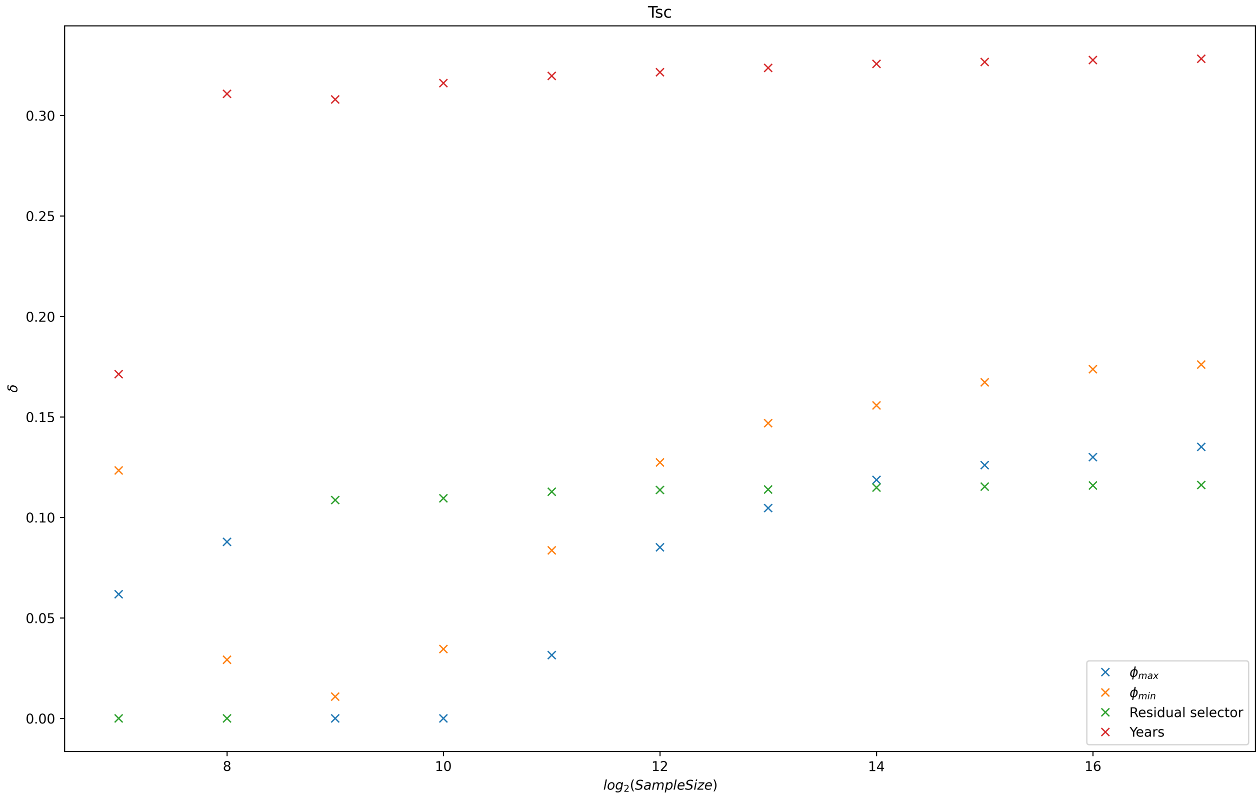

Fig. 17: Convergence plot for Tsc.

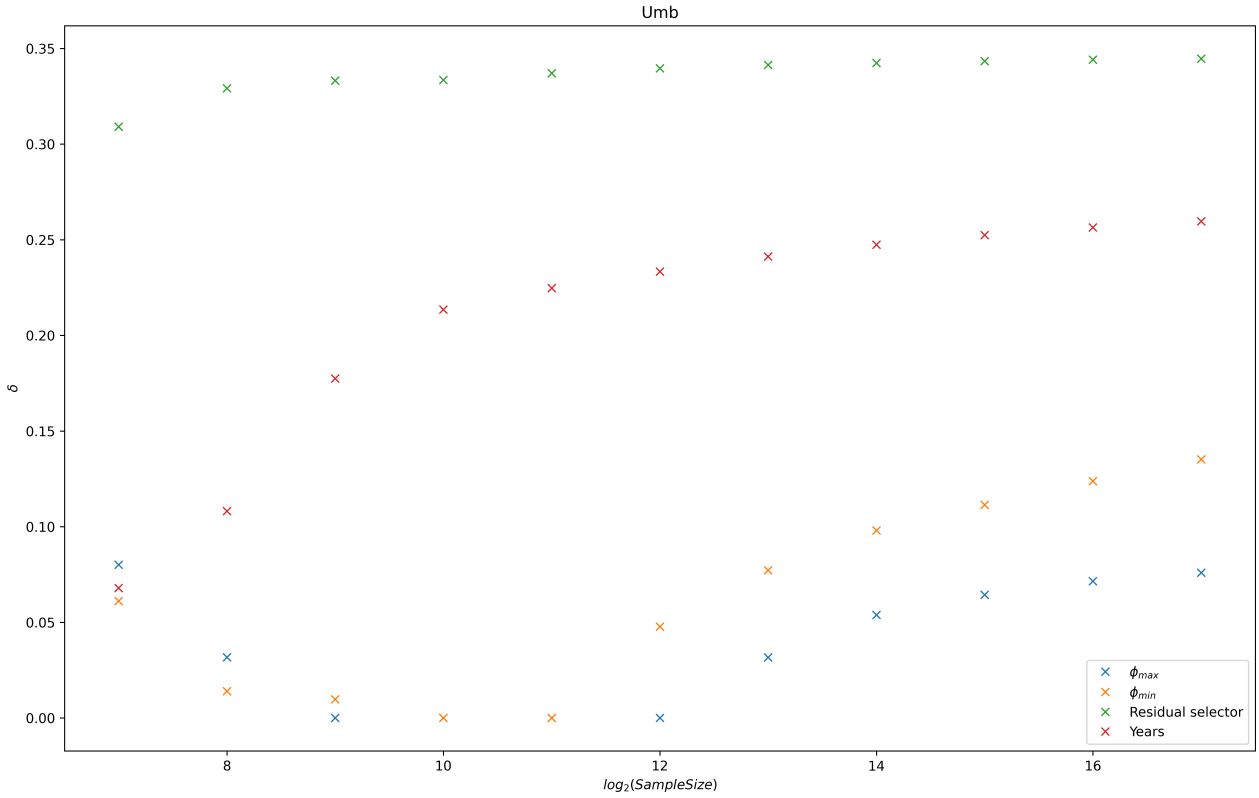

Fig. 18: Convergence plot for Umb.

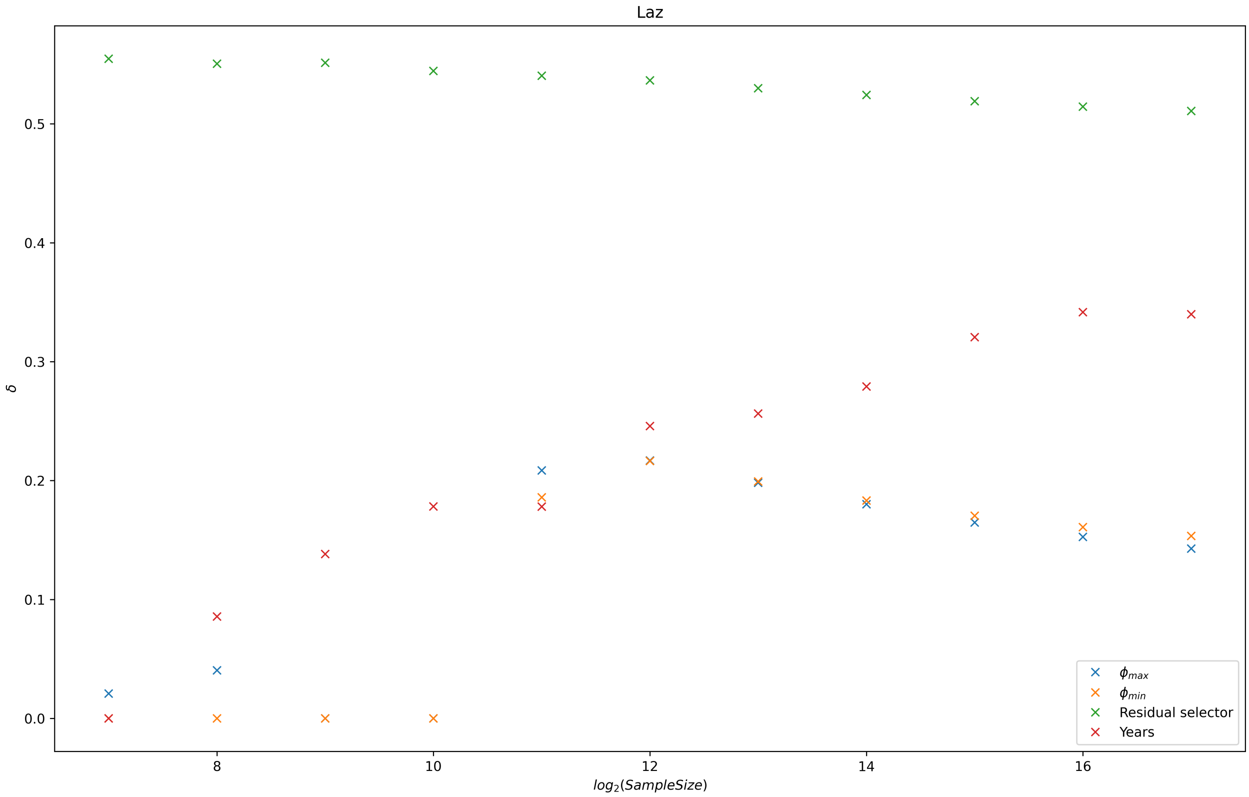

Fig. 19: Convergence plot for Laz.

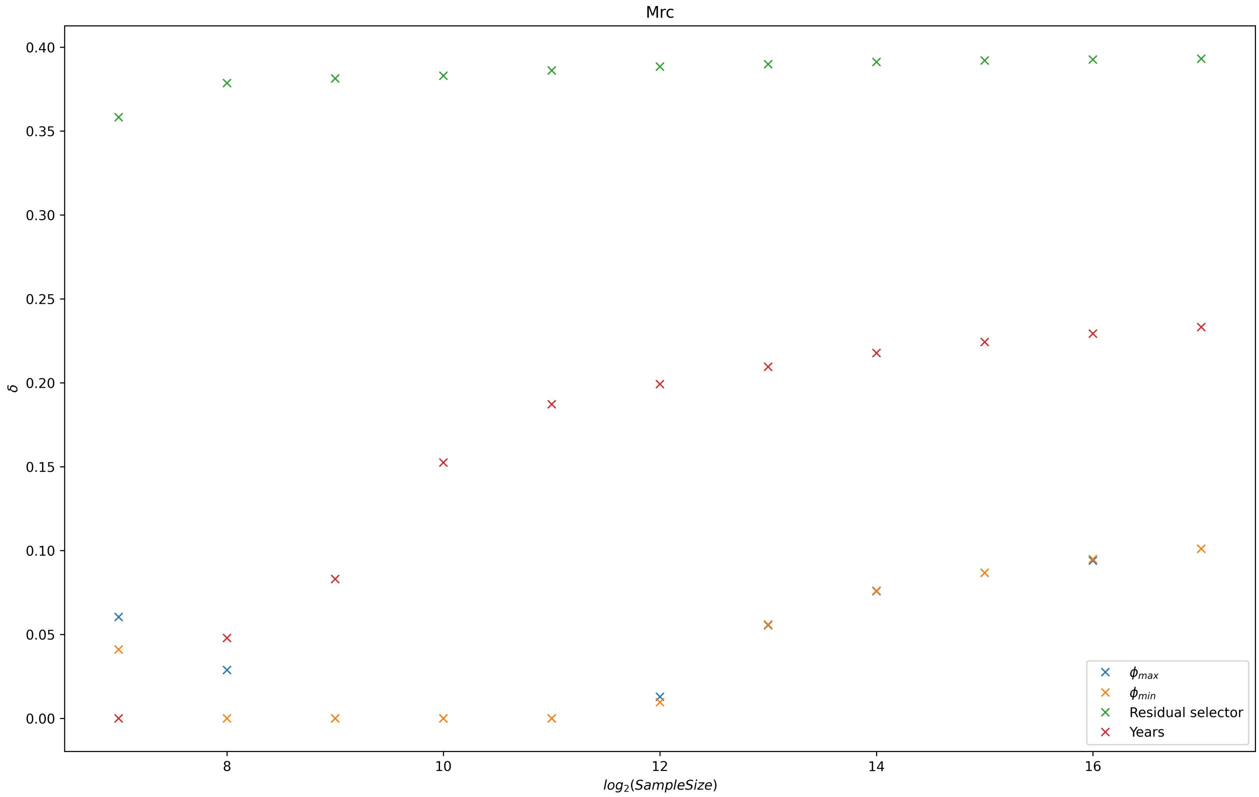

Fig. 20: Convergence plot for Mrc.

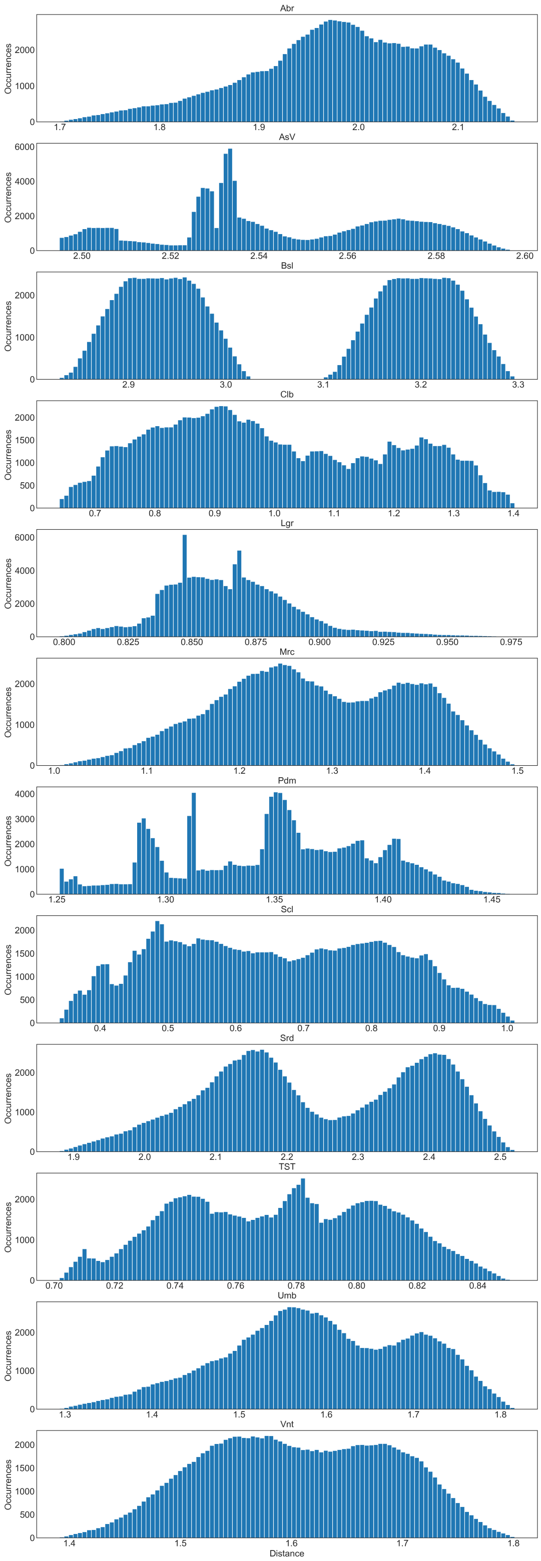

Fig. 21: Distributions for Pdm, AsV, Lgr, Abr, Bsl, Clb, Scl, Srd, TST, Vnt, Umb, Mrc. Number of occurrences in the simulation against cumulative distance between the estimated and reported expenditures

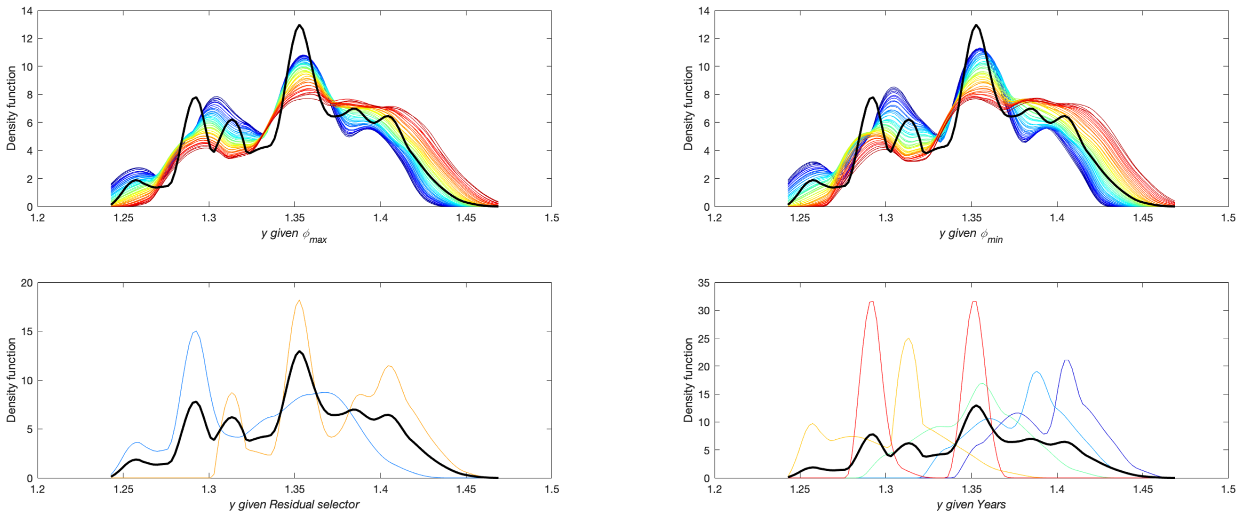

Fig. 22: Conditional output distributions for Pdm when fixing the uncertain input parameters.

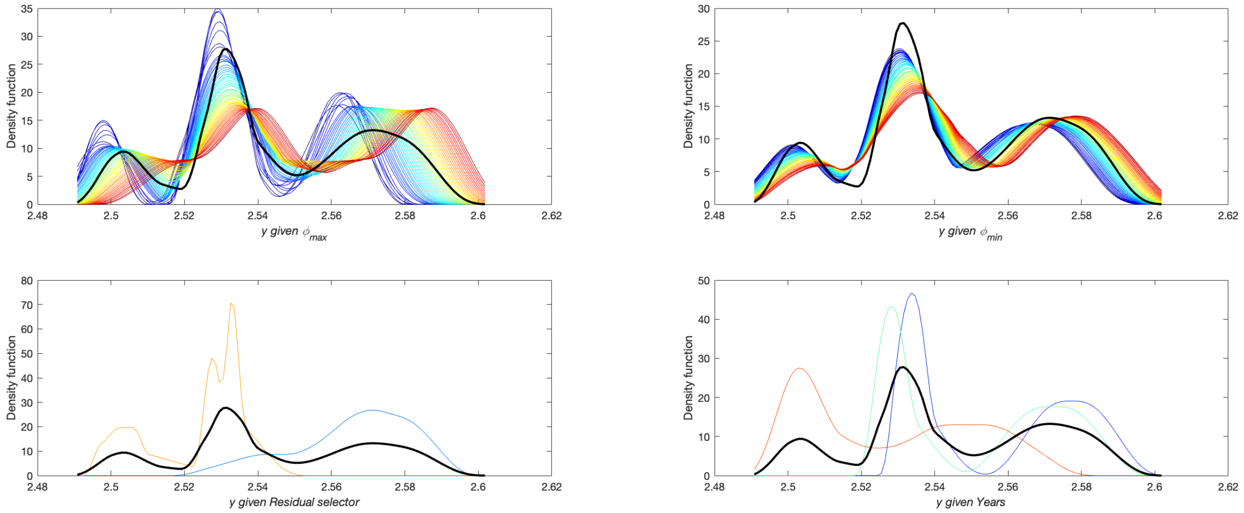

Fig. 23: Conditional output distributions for AsV when fixing the uncertain input parameters.

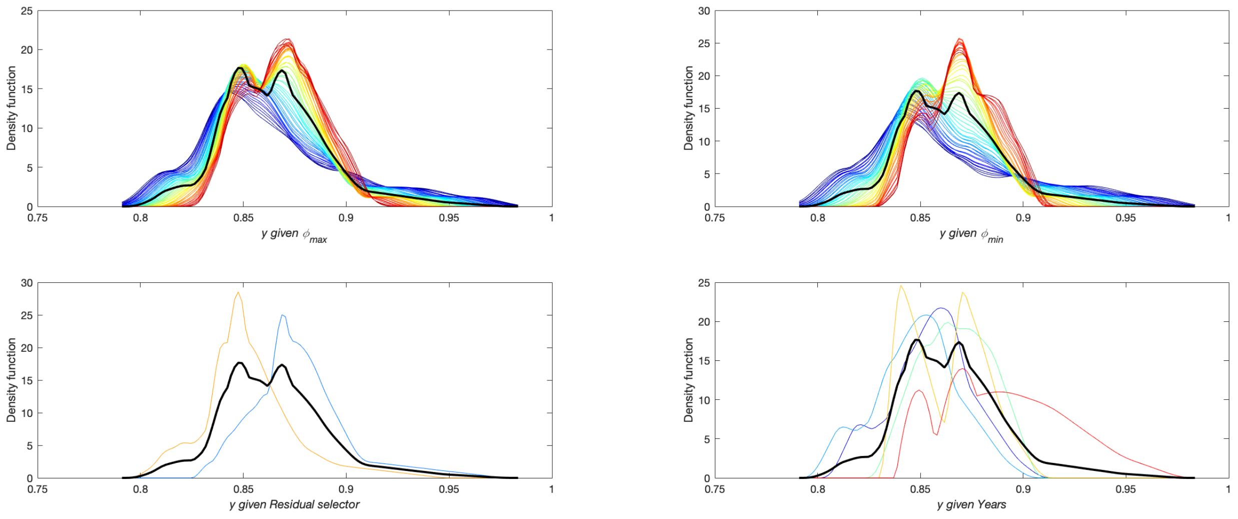

Fig. 24: Conditional output distributions for Lgr when fixing the uncertain input parameters.

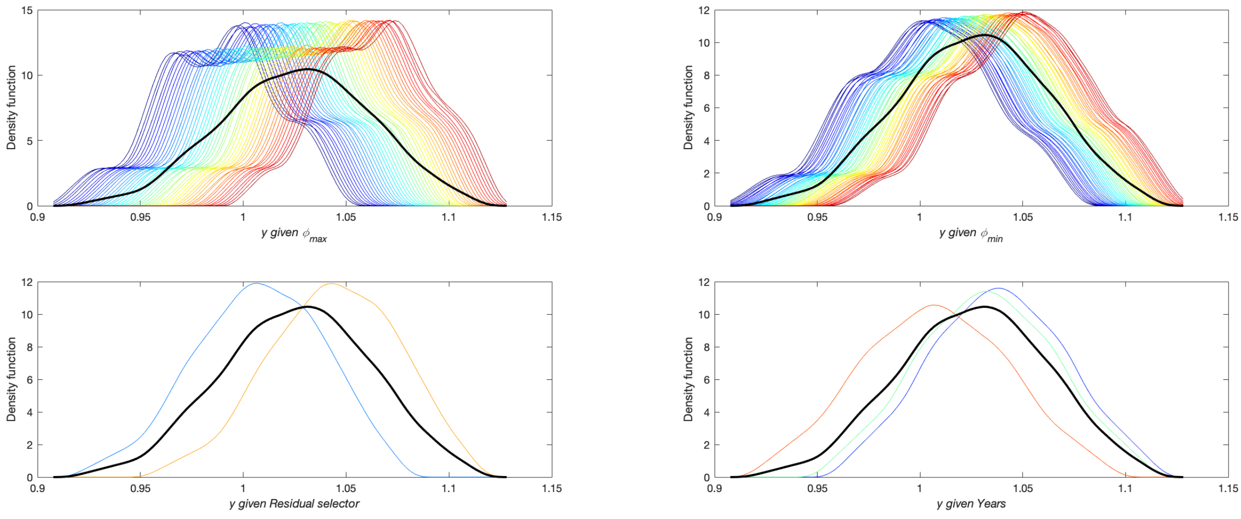

Fig. 25: Conditional output distributions for Lmb when fixing the uncertain input parameters.

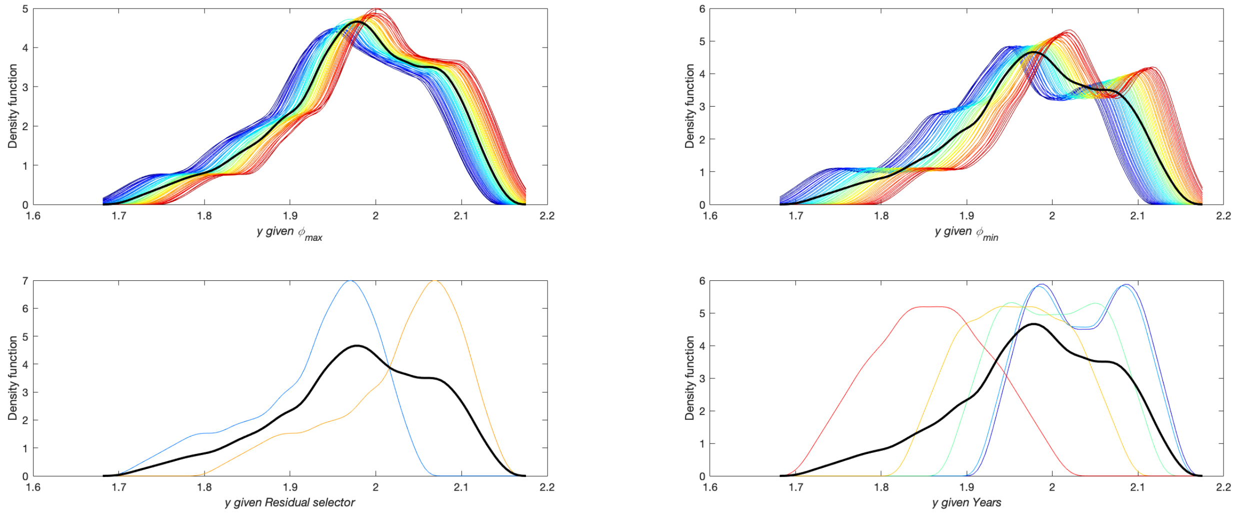

Fig. 26: Conditional output distributions for Abr when fixing the uncertain input parameters.

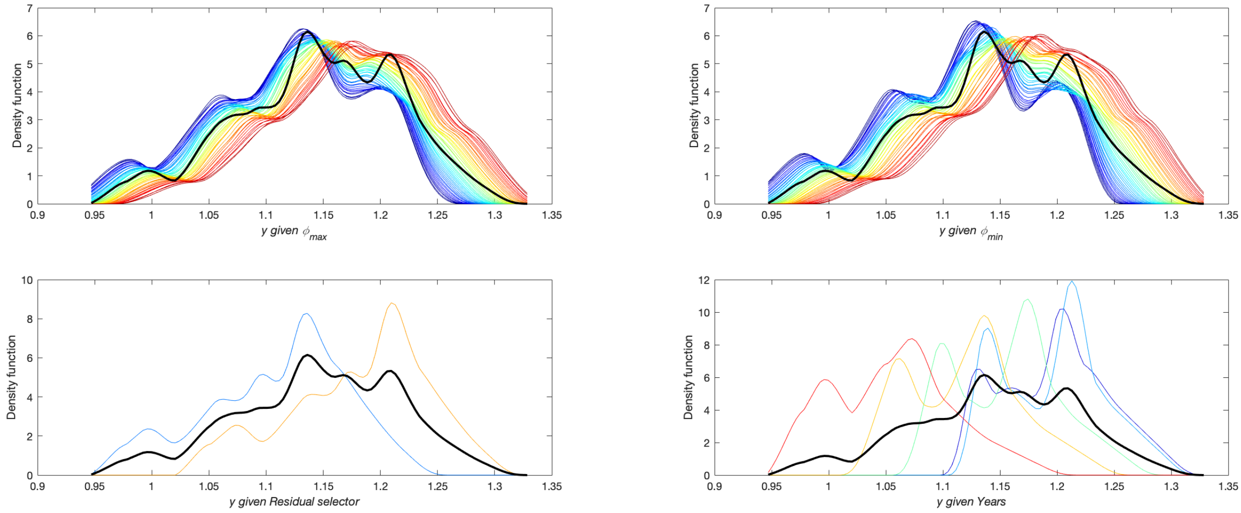

Fig. 27: Conditional output distributions for Mls when fixing the uncertain input parameters.

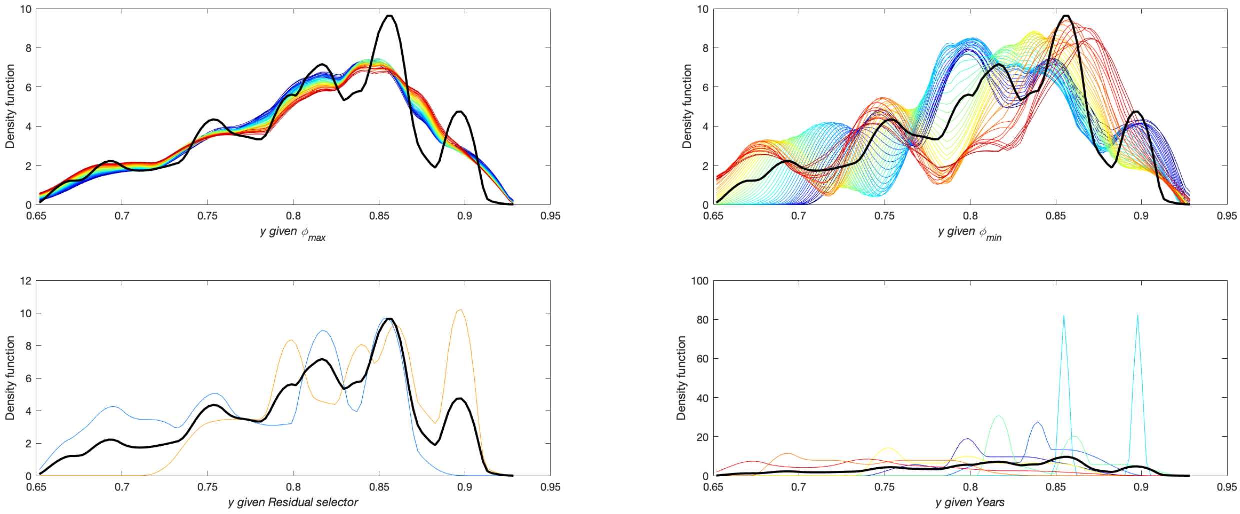

Fig. 28: Conditional output distributions for Cmp when fixing the uncertain input parameters.

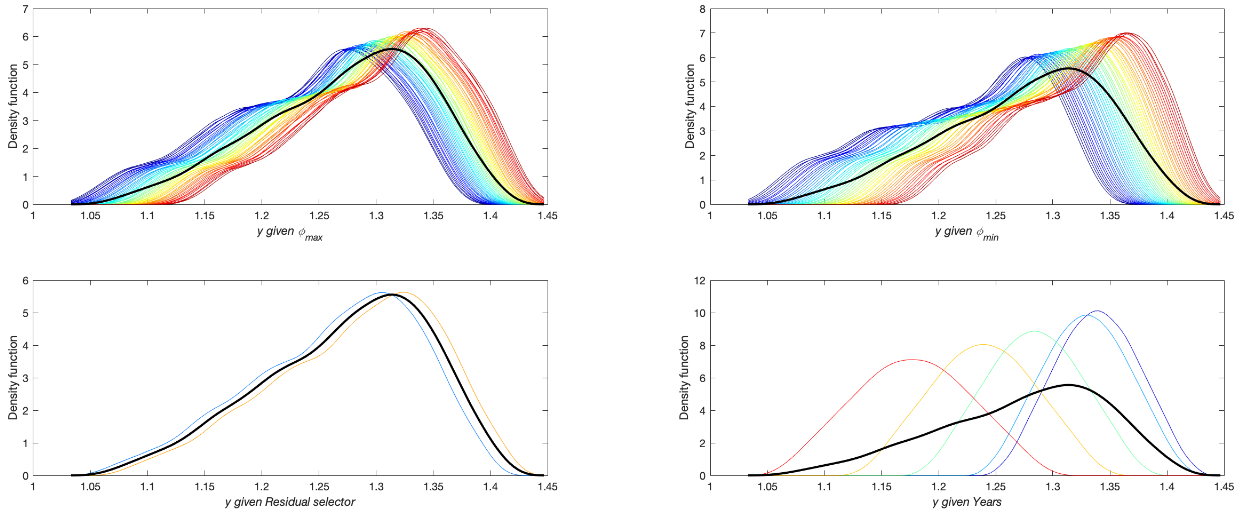

Fig. 29: Conditional output distributions for Apl when fixing the uncertain input parameters.

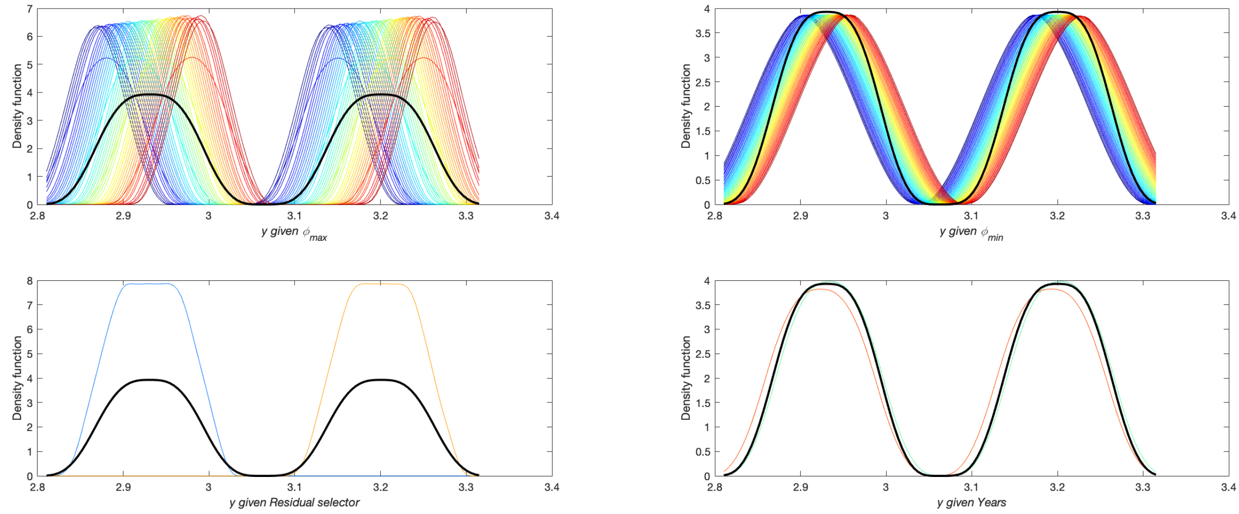

Fig. 30: Conditional output distributions for Bsl when fixing the uncertain input parameters.

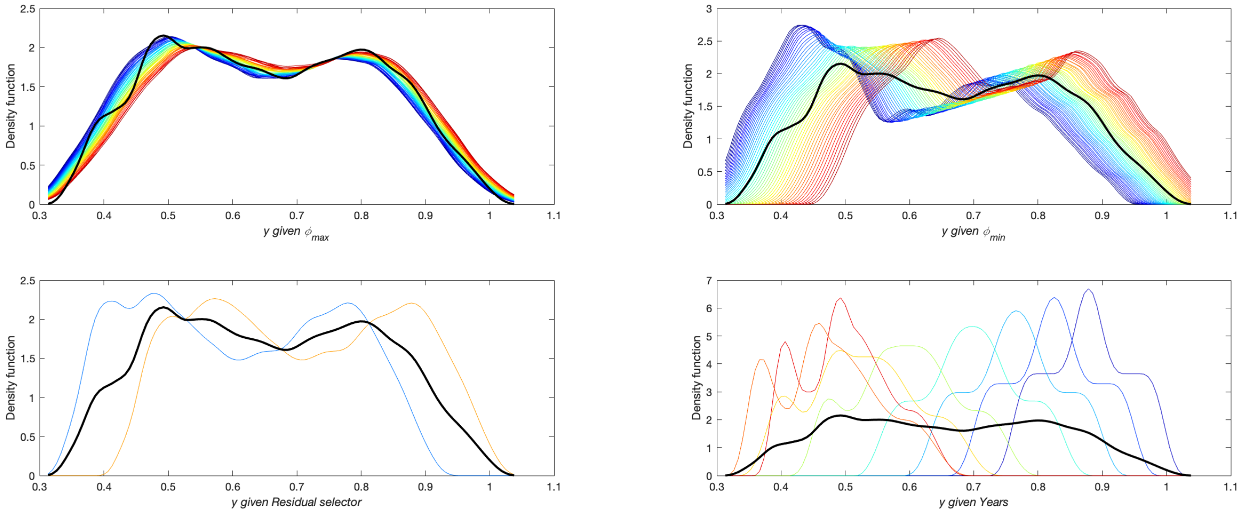

Fig. 31: Conditional output distributions for Scl when fixing the uncertain input parameters.

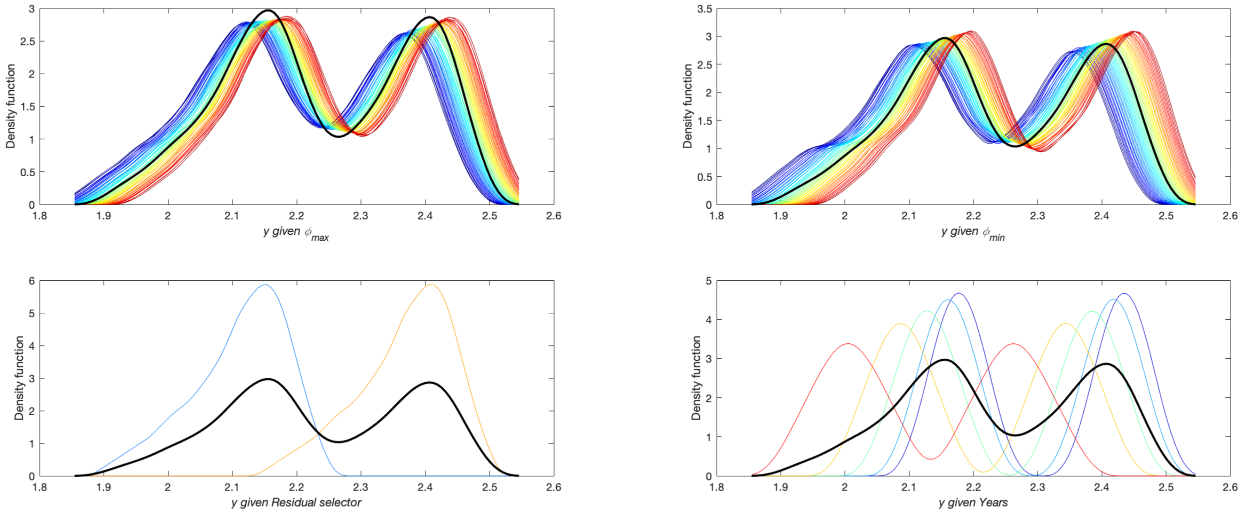

Fig. 32: Conditional output distributions for Srd when fixing the uncertain input parameters.

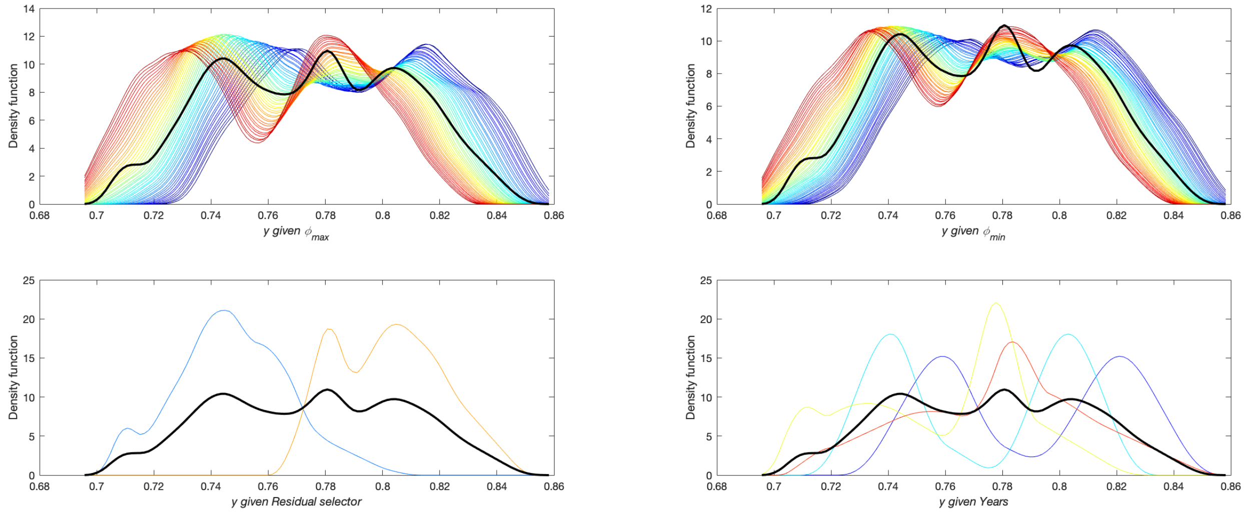

Fig. 33: Conditional output distributions for TST when fixing the uncertain input parameters.

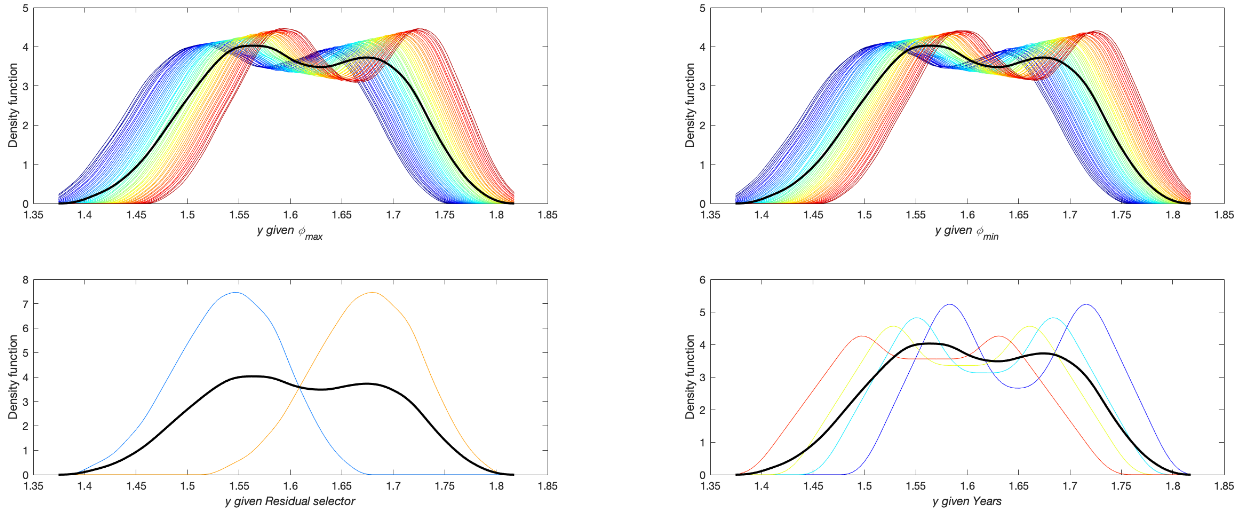

Fig. 34: Conditional output distributions for Vnt when fixing the uncertain input parameters.

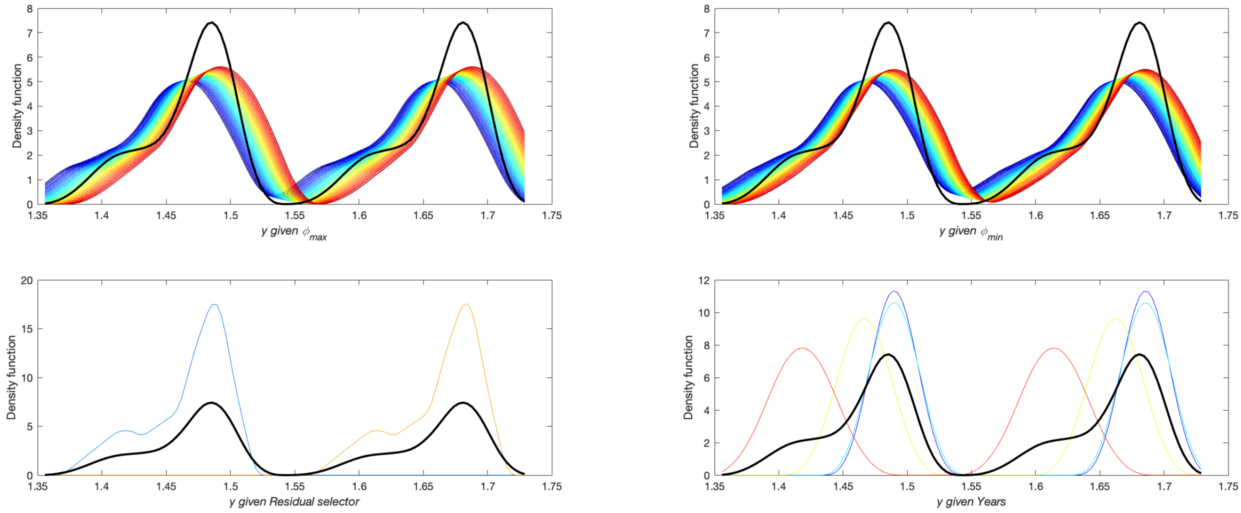

Fig. 35: Conditional output distributions for FVG when fixing the uncertain input parameters.

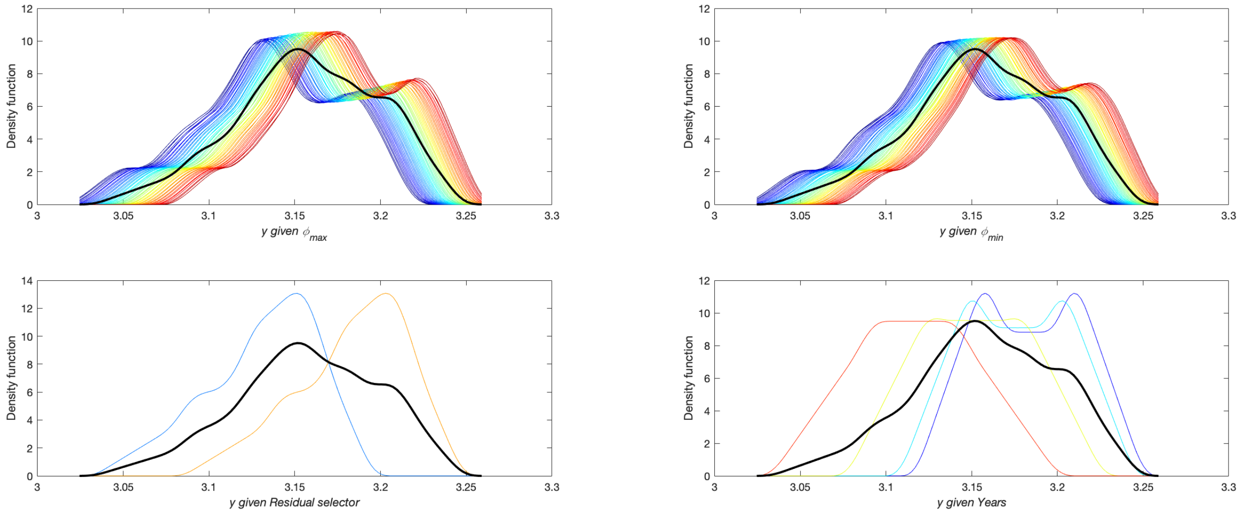

Fig. 36: Conditional output distributions for EmR when fixing the uncertain input parameters.

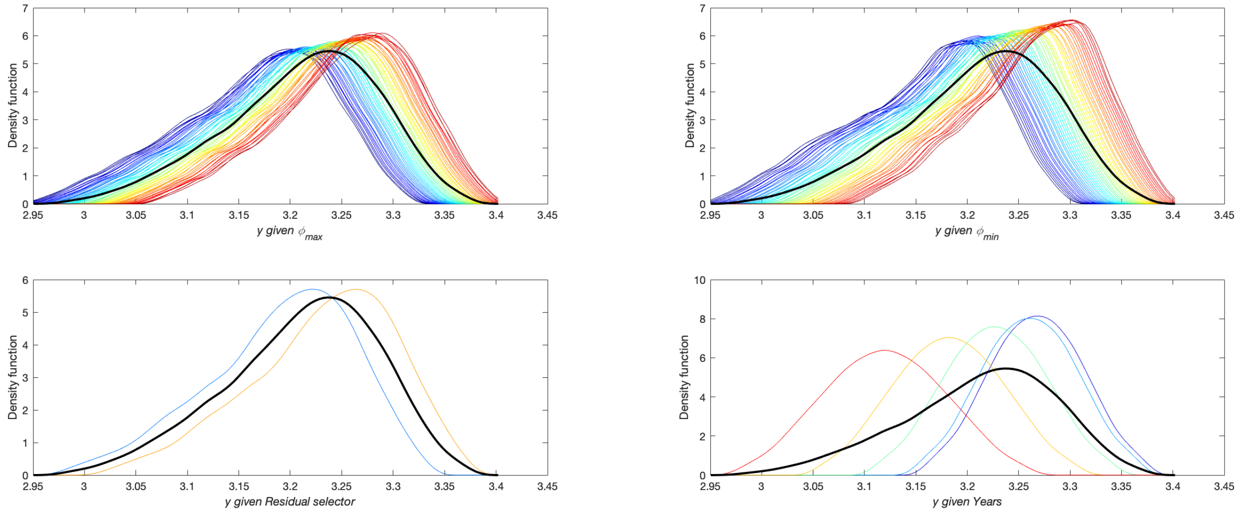

Fig. 37: Conditional output distributions for Tsc when fixing the uncertain input parameters.

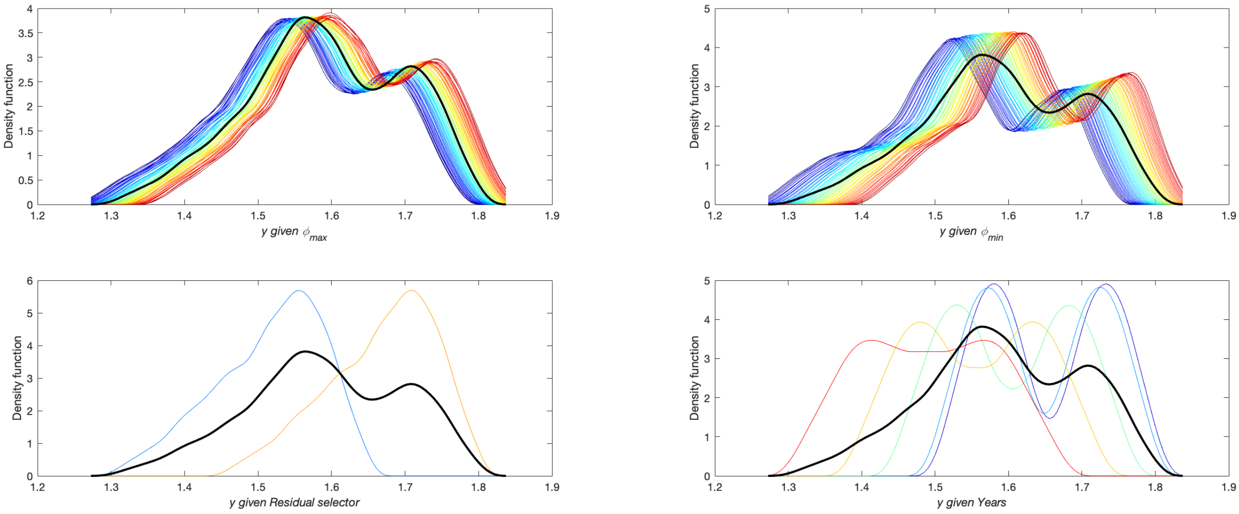

Fig. 38: Conditional output distributions for Umb when fixing the uncertain input parameters.

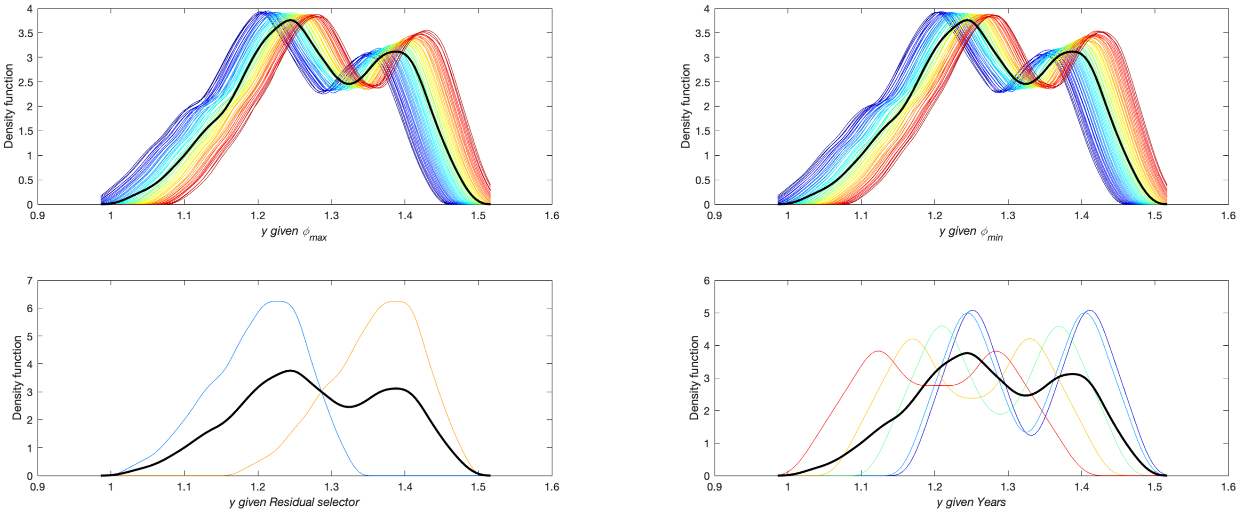

Fig. 39: Conditional output distributions for Mrc when fixing the uncertain input parameters.
