# Supplementary material for: Improving the reliability of cohesion policy databases
Source: PLoS One. 2022 Apr 22;17(4):e0266823. doi: 10.1371/journal.pone.0266823 (PMC9032402; doi:10.1371/journal.pone.0266823)
Supplement: Attachment — Submitted filename: 2022.03.18_rebuttal.pdf [file pone.0266823.s003.pdf]

Response to the reviewers and editor

Dear Prof. Roberto Savona, we thank the reviewer and yourself for your feedback on our submission **PONE-D-22-00671**. Our reply to comments received is found below. The edited passages are in *italics* and the lines at which the changes were implemented in blue.

Reviewer 1

Reviewer Point P 1.1 — The manuscript presents an innovative data-driven model to reconstruct a reliable temporal pattern for time-lagged statistical monetary, applied to ERDF in Italy. I found the study interesting and well written. In addition, the methodology is not only interesting itself, but it presents a novel easy-to-use tool, freely available online. I have only some minor concerns.

Reply: Thank you, we appreciate your remarks. Please find below the reply to the minor concerns that you raised.

Specific comments

Reviewer Point P 1.2 — Specifically, at p. 6, eq. (4), at the LHS there should be μ_p^s instead of μ_p.

Reply: The reviewer correctly pointed to a lack of consistency in the formula at equation 6. Equation 5 was the wrong one, where it should have been μ_p in lieu of μ_p^s. We have amended this.
See the new version of equation 5, at line 129.

Reviewer Point P 1.3 — European Statistics Code of Practice mentioned in the conclusions should be cited and briefly recalled for the benefit of non-expert readers.

Reply: We have included a citation [1] to the code and briefly explained its significance in the economy of the manuscript.
[...][25] where the common quality framework of the European Statistical System for the National Statistical Authorities and Eurostat is defined
Lines 336-338.

Reviewer Point P 1.4 — The authors should explain what are the advantage of using their technique compared to the methodologies applied to obtain regionalized data on Cohesion funds available in the following webpage of the European Commission: `https://cohesiondata.ec.europa.eu/Other/Historic-EU-payments-regionalised-and-modelled/tc55-7ysv`. In particular, at this link `https://ec.europa.eu/regional_policy/en/policy/evaluations/ec/2007-2013/#13` it is reported how data on Cohesion funds at regional level for period 2007-2013 were modelled, and at this link `https://ec.europa.eu/regional_policy/sources/docgener/evaluation/pdf/expost2006/expenditure_final.pdf` the methodology for period 2006-2006 is described.

Reply: The first weblink is to the database which is associated with the technical report (ref. [22] in the manuscript) [2]. In this submission to PLOS ONE that work is advanced because we complete the data until the end of the programming period 2013-2020, we fully and self-consistently define the theoretical framework and we systematise our analysis. [3] (Ref. [13] in the manuscript) is the same as the second weblink, which we used for the regionalisation of the data, that we advance by proposing our approach to retrieve expenses from the pattern of payments. Finally, the third link is to an older approach, which was in turn advanced by the second one [3] (ref. [13] in the manuscript).
The advantages of our methodology are detailed in the abstract and illustrated in Figure 1. Estimating the expenses provides figures closer to those of the Italian authority (validation standard) in comparison with those from the European Commission database which is simply based on the regionalisation procedure of the payments as described in [3] (reference [13] in the manuscript).
We have also included the reference in the first link [4] (as ref. 23 in the manuscript) and elaborated on our contribution to the state of the art.
[...][14, 22, 23] and validate the ERDF figures for Italy against those of the national managing authority
See lines 175-176.

References

1. Commission E. European statistics Code of Practice — revised edition 2017. Tech. rep. 2017. URL: https://ec.europa.eu/eurostat/web/products-catalogues/-/KS-02-18-142 (visited on 03/17/2022).
2. Lo Piano S and Walsh J. Historic EU payments by region: 1988-2018. Tech. rep. 2018CE16BAT115. Brussels: European Commission, 2020:1–40. URL: https://cohesiondata.ec.europa.eu/stories/s/Historic-EU-payments-by-region-1988-2018/47md-x4nq/ (visited on 05/30/2020).
3. Wishlade F, Michie R, Familiari G, Schneiderwind P, and Resch A. Financial Instruments for Enterprises - Final Report : Ex post evaluation of Cohesion Policy programmes 2007 - 2013, focusing on the European Regional Development Fund (ERDF) and the Cohesion Fund (CF). Report. ISBN: 9789279591211 Num Pages: 237. Brussels: European Commission, 2016. URL: https://doi.org/10.2776/259268 (visited on 05/03/2020).
4. Commission E. Historic EU payments - regionalised and modelled — Data — European Structural and Investment Funds. 2020. URL: https://cohesiondata.ec.europa.eu/Other/Historic-EU-payments-regionalised-and-modelled/tc55-7ysv (visited on 03/19/2022).
