# Supplementary material for: Improving the reliability of cohesion policy databases
Source: PLoS One. 2022 Apr 22;17(4):e0266823. doi: 10.1371/journal.pone.0266823 (PMC9032402; doi:10.1371/journal.pone.0266823)
Supplement: Attachment — Submitted filename: Review.docx [file pone.0266823.s002.docx]

The manuscript presents an innovative data-driven model to reconstruct a reliable temporal pattern for time-lagged statistical monetary, applied to ERDF in Italy. I found the study interesting and well written. In addition, the methodology is not only interesting itself, but it presents a novel easy-to-use tool, freely available online.

I have only some minor concerns.

Specifically, at p. 6, eq. (4), at the LHS there should be $\mu_{p}^{s}$ instead of $\mu_{p}$.

European Statistics Code of Practice mentioned in the conclusions should be cited and briefly recalled for the benefit of non-expert readers.

The authors should explain what are the advantage of using their technique compared to the methodologies applied to obtain regionalized data on Cohesion funds available in the following webpage of the European Commission: <https://cohesiondata.ec.europa.eu/Other/Historic-EU-payments-regionalised-and-modelled/tc55-7ysv>. In particular, at this link <https://ec.europa.eu/regional_policy/en/policy/evaluations/ec/2007-2013/#13> it is reported how data on Cohesion funds at regional level for period 2007-2013 were modelled, and at this link <https://ec.europa.eu/regional_policy/sources/docgener/evaluation/pdf/expost2006/expenditure_final.pdf> the methodology for period 2006-2006 is described.
